# Supplementary material for: Membrane-Active Macromolecules Resensitize NDM-1 Gram-Negative Clinical Isolates to Tetracycline Antibiotics
Source: PLoS One. 2015 Mar 19;10(3):e0119422. doi: 10.1371/journal.pone.0119422 (PMC4366164; doi:10.1371/journal.pone.0119422)
Supplement: S1 Checklist — (PDF) (PDF) [file pone.0119422.s001.pdf]

The ARRIVE Guidelines Checklist

Animal Research: Reporting In Vivo Experiments

Carol Kilkenny[1], William J Browne[2], Innes C Cuthill[3], Michael Emerson[4] and Douglas G Altman[5]

[1]The National Centre for the Replacement, Refinement and Reduction of Animals in Research, London, UK, [2]School of Veterinary Science, University of Bristol, Bristol, UK, [3]School of Biological Sciences, University of Bristol, Bristol, UK, [4]National Heart and Lung Institute, Imperial College London, UK, [5]Centre for Statistics in Medicine, University of Oxford, Oxford, UK.

	ITEM	RECOMMENDATION	Section/ Paragraph
Title	1	Provide as accurate and concise a description of the content of the article as possible.	See the attachment
Abstract	2	Provide an accurate summary of the background, research objectives, including details of the species or strain of animal used, key methods, principal findings and conclusions of the study.	See the attachment
**INTRODUCTION**			
Background	3	a. Include sufficient scientific background (including relevant references to previous work) to understand the motivation and context for the study, and explain the experimental approach and rationale.<br>b. Explain how and why the animal species and model being used can address the scientific objectives and, where appropriate, the study's relevance to human biology.	See the attachment
Objectives	4	Clearly describe the primary and any secondary objectives of the study, or specific hypotheses being tested.	See the attachment
**METHODS**			
Ethical statement	5	Indicate the nature of the ethical review permissions, relevant licences (e.g. Animal [Scientific Procedures] Act 1986), and national or institutional guidelines for the care and use of animals, that cover the research.	Materials and Methods
Study design	6	For each experiment, give brief details of the study design including:<br>a. The number of experimental and control groups.<br>b. Any steps taken to minimise the effects of subjective bias when allocating animals to treatment (e.g. randomisation procedure) and when assessing results (e.g. if done, describe who was blinded and when).<br>c. The experimental unit (e.g. a single animal, group or cage of animals).<br>A time-line diagram or flow chart can be useful to illustrate how complex study designs were carried out.	Materials and Methods
Experimental procedures	7	For each experiment and each experimental group, including controls, provide precise details of all procedures carried out. For example:<br>a. How (e.g. drug formulation and dose, site and route of administration, anaesthesia and analgesia used [including monitoring], surgical procedure, method of euthanasia). Provide details of any specialist equipment used, including supplier(s).<br>b. When (e.g. time of day).<br>c. Where (e.g. home cage, laboratory, water maze).<br>d. Why (e.g. rationale for choice of specific anaesthetic, route of administration, drug dose used).	Materials and Methods<br>All the procedures comply with the OECD guidelines (OECD 425)
Experimental animals	8	a. Provide details of the animals used, including species, strain, sex, developmental stage (e.g. mean or median age plus age range) and weight (e.g. mean or median weight plus weight range).<br>b. Provide further relevant information such as the source of animals, international strain nomenclature, genetic modification status (e.g. knock-out or transgenic), genotype, health/immune status, drug or test naïve, previous procedures, etc.	Materials and Methods

Housing and husbandry	9	Provide details of:<br>a. Housing (type of facility e.g. specific pathogen free [SPF]; type of cage or housing; bedding material; number of cage companions; tank shape and material etc. for fish).<br>b. Husbandry conditions (e.g. breeding programme, light/dark cycle, temperature, quality of water etc for fish, type of food, access to food and water, environmental enrichment).<br>c. Welfare-related assessments and interventions that were carried out prior to, during, or after the experiment.	Materials and Methods
Sample size	10	a. Specify the total number of animals used in each experiment, and the number of animals in each experimental group.<br>b. Explain how the number of animals was arrived at. Provide details of any sample size calculation used.<br>c. Indicate the number of independent replications of each experiment, if relevant.	Materials and Methods<br>Comply with the OECD guidelines (OECD 425)
Allocating animals to experimental groups	11	a. Give full details of how animals were allocated to experimental groups, including randomisation or matching if done.<br>b. Describe the order in which the animals in the different experimental groups were treated and assessed.	Materials and Methods<br>Comply with the OECD guidelines (OECD 425)
Experimental outcomes	12	Clearly define the primary and secondary experimental outcomes assessed (e.g. cell death, molecular markers, behavioural changes).	Results and Discussion
Statistical methods	13	a. Provide details of the statistical methods used for each analysis.<br>b. Specify the unit of analysis for each dataset (e.g. single animal, group of animals, single neuron).<br>c. Describe any methods used to assess whether the data met the assumptions of the statistical approach.	Materials and Methods
RESULTS			
Baseline data	14	For each experimental group, report relevant characteristics and health status of animals (e.g. weight, microbiological status, and drug or test naïve) prior to treatment or testing. (This information can often be tabulated).	See the attachment
Numbers analysed	15	a. Report the number of animals in each group included in each analysis. Report absolute numbers (e.g. 10/20, not 50%[2]).<br>b. If any animals or data were not included in the analysis, explain why.	See the attachment
Outcomes and estimation	16	Report the results for each analysis carried out, with a measure of precision (e.g. standard error or confidence interval).	Results and Discussion, Figures and Tables
Adverse events	17	a. Give details of all important adverse events in each experimental group.<br>b. Describe any modifications to the experimental protocols made to reduce adverse events.	See the attachment
DISCUSSION			
Interpretation/ scientific implications	18	a. Interpret the results, taking into account the study objectives and hypotheses, current theory and other relevant studies in the literature.<br>b. Comment on the study limitations including any potential sources of bias, any limitations of the animal model, and the imprecision associated with the results[2].<br>c. Describe any implications of your experimental methods or findings for the replacement, refinement or reduction (the 3Rs) of the use of animals in research.	See the attachment

Generalisability/ translation	19	Comment on whether, and how, the findings of this study are likely to translate to other species or systems, including any relevance to human biology.	See the attachment
Funding	20	List all funding sources (including grant number) and the role of the funder(s) in the study.	See the attachmen t

References:
1. Kilkenny C, Browne WJ, Cuthill IC, Emerson M, Altman DG (2010) Improving Bioscience Research Reporting: The ARRIVE Guidelines for Reporting Animal Research. *PLoS Biol* 8(6): e1000412. doi:10.1371/journal.pbio.1000412
2. Schulz KF, Altman DG, Moher D, the CONSORT Group (2010) CONSORT 2010 Statement: updated guidelines for reporting parallel group randomised trials. *BMJ* 340:c332.

Attachment to The ARRIVE Guidelines Checklist:

Item 1: Membrane-active Macromolecules Resensitize NDM-1 Gram-negative Clinical Isolates to Tetracycline Antibiotics

Item 2: The study aimed at finding a treatment of Gram-negative bacterial infections caused by NDM-1 producing pathogens. The combination of membrane-active macromolecules (MAMs) and tetracycline antibiotics was proposed for the treatment. First, the acute toxicity of MAMs alone and in combination with antibiotics was tested in Female Balb/c mice (6-8 weeks, 18-22 g) using single-dose acute toxicity studies with different routes of administration (intravenous (i.v.) (tail vein), intraperitoneal (i.p.) and subcutaneous (s.c.) (over the flank)) and sub-chronic toxicity studies. It was found that MAMs alone and in combination with the antibiotic were found to be safe for performing the studies in mice. Next, antibacterial efficacy studies were performed in mice using Neutropenic Thigh Infection model. The study showed that combination with antibiotics had significantly decreased bacterial burden compared to the untreated mice.

Item 3: a) Bacterial infections caused by multi-drug resistant Gram-negative bacteria have become a major threat to the global public health. The study aimed at providing a combination approach of MAMs and tetracycline antibiotics for the treatment of Gram-negative bacterial infections caused by NDM-1 producing pathogens. Towards this, first, the toxicity of the test drugs needed to be performed in mice to determine the maximum tolerable dose. Then, the antibacterial efficacy studies were performed in mice for determining the efficacy of the combination in-vivo.

b) Mice models can more or less be used to understand the bacterial infections and can be extended to humans. In the present study, we used routes of administration in mice like intravenous, intraperitoneal and subcutaneous that can also be performed in humans for administering the drugs. Also, the mouse thigh infection model is generally used to mimic a tissue infection caused by bacteria in humans.

Item 4: The primary objective of the study was to find a treatment option for Gram-negative bacterial infections caused by NDM-1 producing pathogens. The secondary objective was to find out whether the combination of the MAMs and tetracycline antibiotics would be more efficacious than the MAMs or antibiotics alone using the mice models.

Item 5: Animal studies were performed according to the protocols approved by Institutional Animal Ethics Committee (IAEC) of Jawaharlal Nehru Centre for Advanced Scientific Research (JNCASR) and National Institute of Veterinary Epidemiology and Disease Informatics (NIVEDI). Acute and sub-chronic toxicity studies were performed at Jawaharlal Nehru Centre for Advanced Scientific Research (JNCASR), Bengaluru (CPCSEA/201) in accordance with institutional ethical guidelines. Infection studies were performed at National Institute of Veterinary Epidemiology and Disease Informatics (NIVEDI). The animal experiments were approved by the Institutional Animal Ethics Committee (IAEC) of National Institute of Veterinary Epidemiology and Disease Informatics (NIVEDI), Bengaluru (881/GO/ac/05/CPCSEA) and carried out as per the guidelines of Committee for the purpose of Supervision and Experiments on Animals (CPCSEA), Ministry of Environment and Forests, New Delhi, India.

Item 6: a) For the systemic toxicity studies 10 experimental groups and 3 control groups were used. For sub-chronic toxicity studies, 4 experimental groups and 1 control group were used. For the infection studies, three experimental groups and one control group was used.

b) Animals were randomly selected, marked to permit individual identification and kept in their cages for at least 5 days before the experiment to allow for acclimatization to the experimental conditions.

c) The experiments were performed in individually ventilated cages (IVC) maintained with controlled environment as per the standards

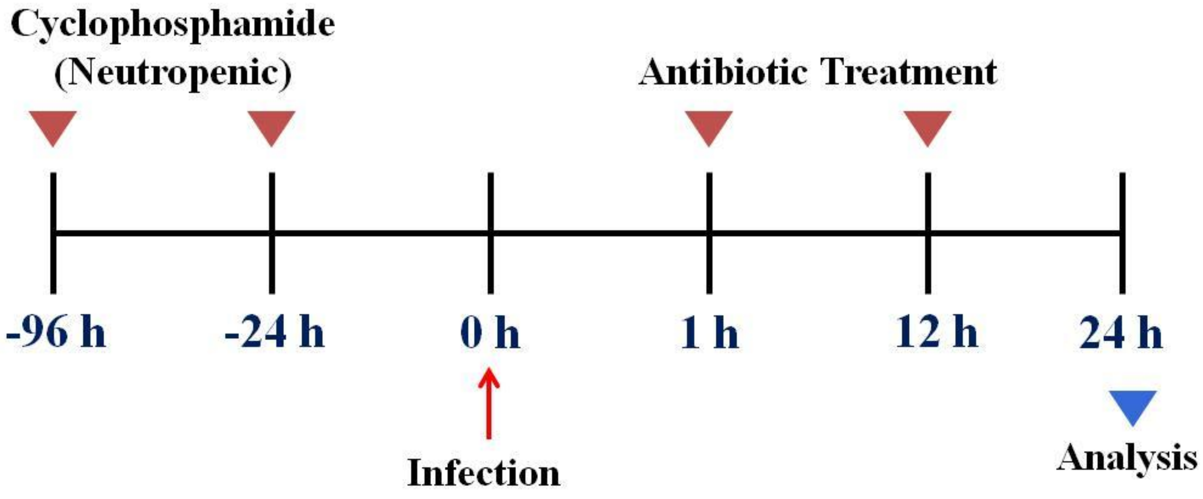

Item 7-11: Pease see Materials and Methods section of the manuscript. All the procedures comply with the OECD guidelines (OECD 425).

Item 12: Please see Results and discussion section of the manuscript.

Item 13: Please see Materials and Methods section of the manuscript.

Item 14: All the mice in the experimental groups were of the age of 6-8 weeks with an average body weight of 18-22 g prior to treatment or testing

Item 15: All the animals in each group were included in each analysis.

Item 16: Please see Results and discussion section of the manuscript.

Item 17: a) The mice in the high dose group (175 mg kg^{-1}) in systemic toxicity studies immediately post-injection of the drug showed clinical signs of tremors, recumbency, sever distress and convulsions, which were indicative of the impending death or moribund condition.

b) For the intraperitoneal (i.p.) and subcutaneous (s.c.) (over the flank) routes of administration, the high dose (175 mg kg^{-1}) was not injected to reduce the animal lethality.

Item 18: a) Please see Results and discussion section of the manuscript.

b) Mice models are used to mimic the conditions of the human pathology and disease. In our methods, before the bacterial infection, mice have to be rendered neutropenic for the infection to occur in mice. This step is needed as mice have stronger immune system than humans. This is a major limitation of mice studies for bacterial infections. C) Use of humanized mice can be a replacement for studying the bacterial infections in mice that might be correlated well with the human disease conditions.

Item 19: The approach of using combination of two drugs for the treatment of bacterial infections was tested in mice models and can very well be translated to other species. As infections are universal, it can be translated in veterinary medicine for other animals and as well as in human medicine.

Item 20: This work was supported by the grant "Ramanujan fellowship" [SR/S2/RJN-43/2009] from the Department of Science and Technology (DST), Govt. of India. The funders had no role in study design, data collection and analysis, decision to publish, or preparation of the manuscript.
