# Supplementary material for: Membrane-Active Macromolecules Resensitize NDM-1 Gram-Negative Clinical Isolates to Tetracycline Antibiotics
Source: PLoS One. 2015 Mar 19;10(3):e0119422. doi: 10.1371/journal.pone.0119422 (PMC4366164; doi:10.1371/journal.pone.0119422)
Supplement: S1 Text — Figure A. Antibacterial activity of minocycline and MAMs. The combination of MAM1 and minocycline (25 μg mL-1 + 3.1 μg mL-1) showed synergistic bactericidal activity whereas MAM1 (25 μg mL-1) alone and minocycline alone (3.1 μg mL-1) were devoid of antibacterial activity against NDM-1 producing E. coli R3336. 20 μL of the bacterial suspension was taken and plated on MacConkey agar plates. Images were taken after 24 h incubation. Figure B. Development of drug-resistance in tetracycline sensitive E. coli (ATCC 25922). Fold of increase in MICs of MAMs, tetracycline alone and in combination with MAMs after exposure of E. coli (ATCC 25922) to sub-MIC concentrations over 32 passages. E. coli did not develop resistance to MAMs. More importantly, resistance against tetracycline alone developed very rapidly but did not develop in presence of MAM1. The MICs of individual agents were as follows: tetracycline (1.56 μg mL-1); MAM1 (15.6 μg mL-1), MAM1 + tetracycline (8 + 1) μg mL-1; MAM2 (250 μg mL-1); MAM2 + tetracycline (62.5 + 1) μg mL-1. Figure C. Mechanistic studies using tetracycline sensitive E. coli (ATCC 25922). (A) E. coli had much faster and higher uptake of tetracycline in presence MAM2 (20, 30, 60 and 100 μg mL-1) than tetracycline alone (100 μg mL-1). (B) MAMs dissipated the membrane potential in E. coli as seen in the increase in fluorescence of DiSC3(5) after addition of MAMs at 25 μg mL-1; (C) Colistin and MAM2 (both at 60 μg mL-1) increased whereas carbonyl cyanide m-chlorophenyl hydrazone, CCCP (5 μg mL-1) decreased the uptake of tetracycline (100 μg mL-1). (D) MAMs caused cytoplasmic membrane permeabilization as seen in the increase in fluorescence of propidium iodide (PI) after addition of MAMs at 25 μg mL-1; Relative fluorescence was calculated by subtracting the fluorescence without the bacteria from the fluorescence of bacteria containing samples. Figure D. Uptake of tetracycline against bla NDM-1 E. coli R3336. (A) Uptake of tetracycline (100 μg mL-1) in [file pone.0119422.s002.doc]

**Supporting Information**

**Membrane-active Macromolecules Re-sensitize NDM-1 Gram-negative Clinical Isolates to Tetracycline Antibiotics**

Divakara S. S. M. Uppu^a^, Goutham B. Manjunath^a^, Venkateswarlu Yarlagadda^a^, Jyothi E. Kaviyil^b^, Raju Ravikumar^b^, Krishnamoorthy Paramanandham^c^, Bibek R. Shome^c^, and Jayanta Haldar^a,*^

^a^Chemical Biology & Medicinal Chemistry Laboratory, New Chemistry Unit, Jawaharlal Nehru Centre for Advanced Scientific Research (JNCASR), Jakkur, Bangalore 560064, India; ^b^Department of Neuromicrobiology, National Institute of Mental Health and Neuro Sciences (NIMHANS), Hosur Road, Bangalore 560029, India; ^c^National Institute of Veterinary Epidemiology and Disease Informatics (NIVEDI), Hebbal, Bengaluru 560024, Karnataka, India

^*^Corresponding author. E-mail: [jayanta@jncasr.ac.in](mailto:jayanta@jncasr.ac.in); Fax: +91-80-2208-2627; Telephone: +91- 80-2208-2565.

**
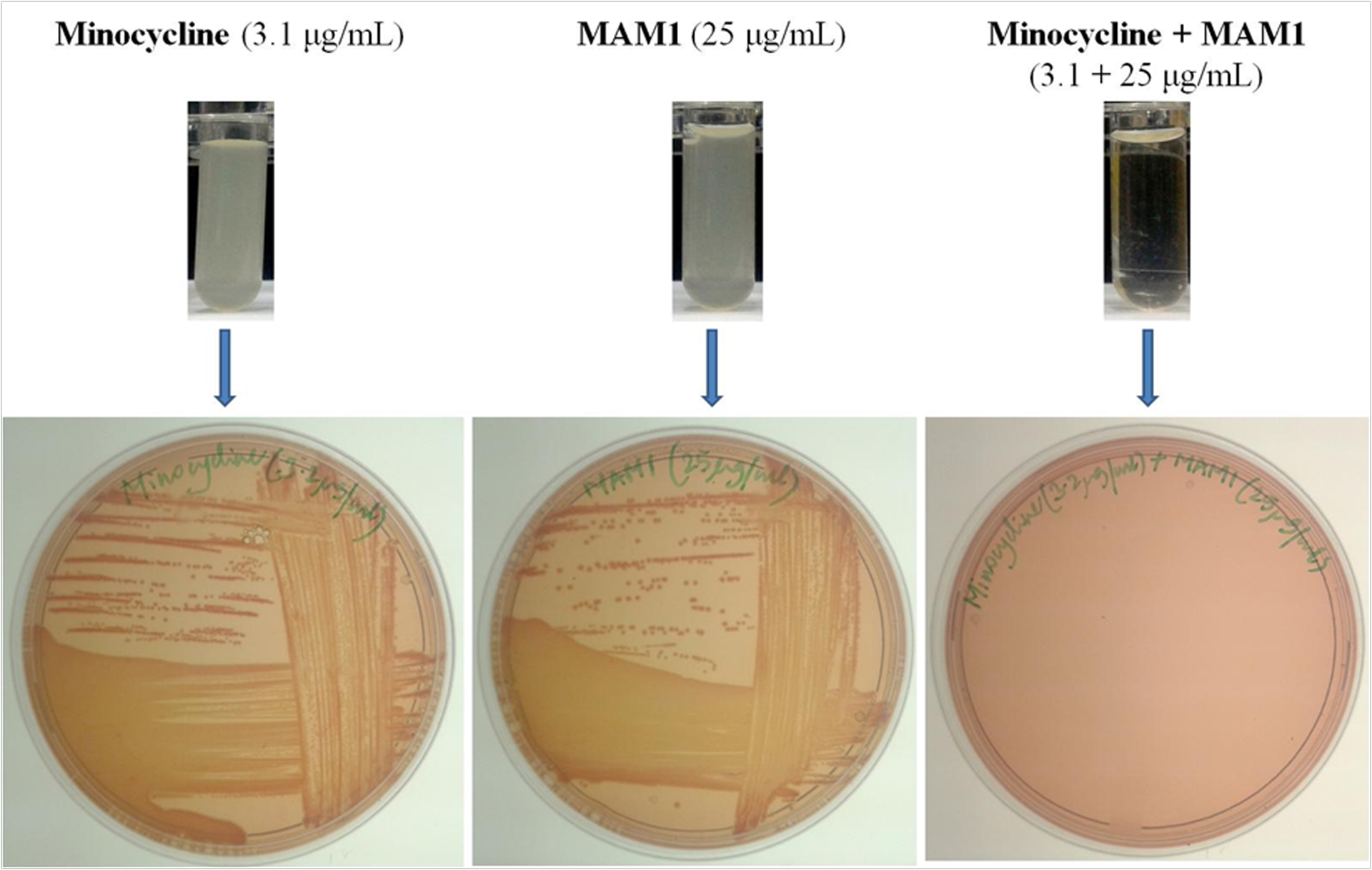
**

**Figure A. Antibacterial activity of minocycline and MAMs.** The combination of MAM1 and minocycline (25 µg mL^-1^ + 3.1 µg mL^-1^) showed synergistic bactericidal activity whereas MAM1 (25 µg mL^-1^) alone and minocycline alone (3.1 µg mL^-1^) were devoid of antibacterial activity against NDM-1 producing *E. coli* R3336. 20 μL of the bacterial suspension was taken and plated on MacConkey agar plates. Images were taken after 24 h incubation.

**
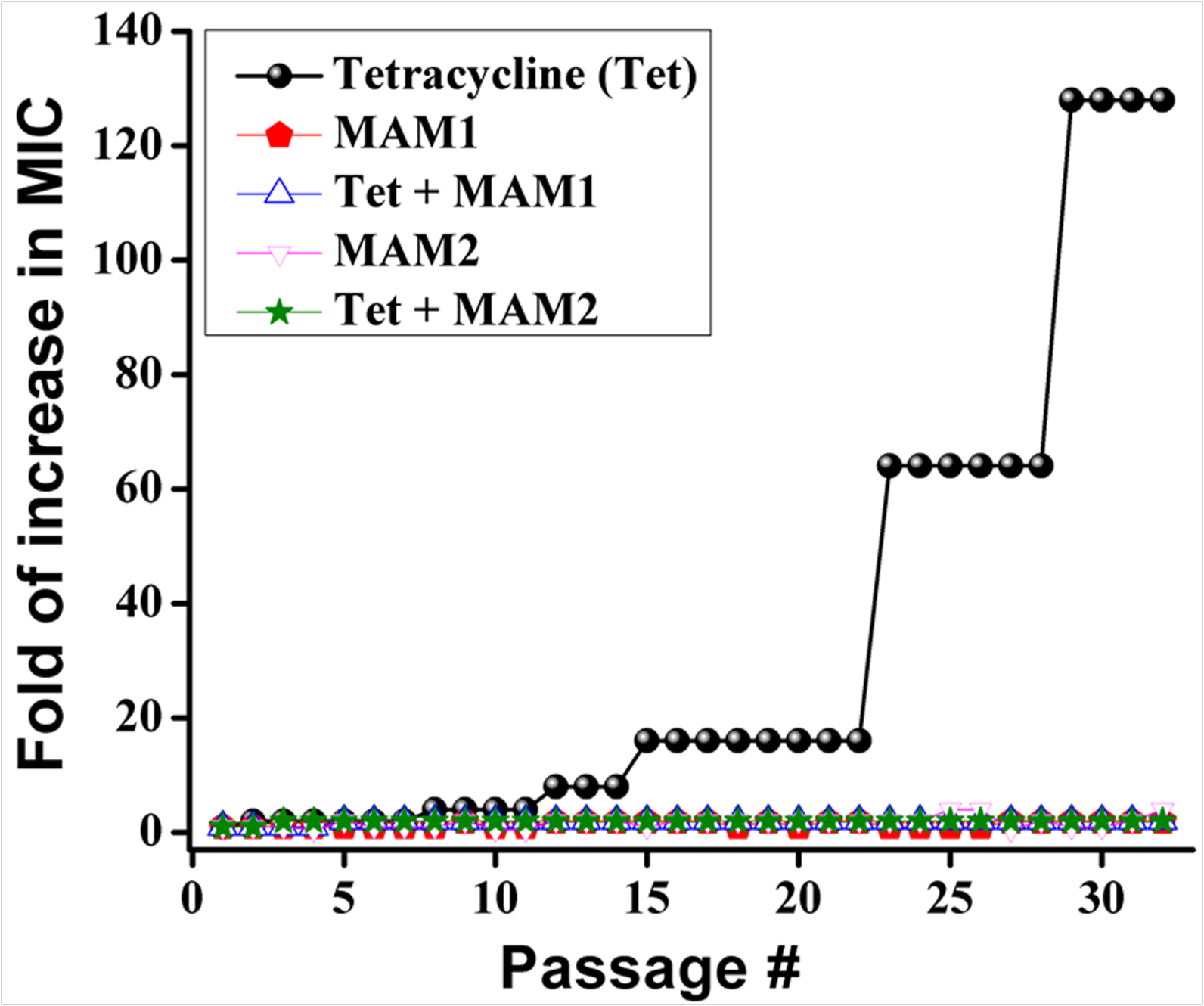
**

**Figure B.** **Development of drug-resistance in tetracycline sensitive *E. coli* (ATCC 25922).** Fold of increase in MICs of MAMs, tetracycline alone and in combination with MAMs after exposure of *E. coli* (ATCC 25922) to sub-MIC concentrations over 32 passages. *E. coli* did not develop resistance to MAMs. More importantly, resistance against tetracycline alone developed very rapidly but did not develop in presence of MAM1. The MICs of individual agents were as follows: tetracycline (1.56 µg mL^-1^); MAM1 (15.6 µg mL^-1^), MAM1 + tetracycline (8 + 1) µg mL^-1^; MAM2 (250 µg mL^-1^); MAM2 + tetracycline (62.5 + 1) µg mL^-1^.

**
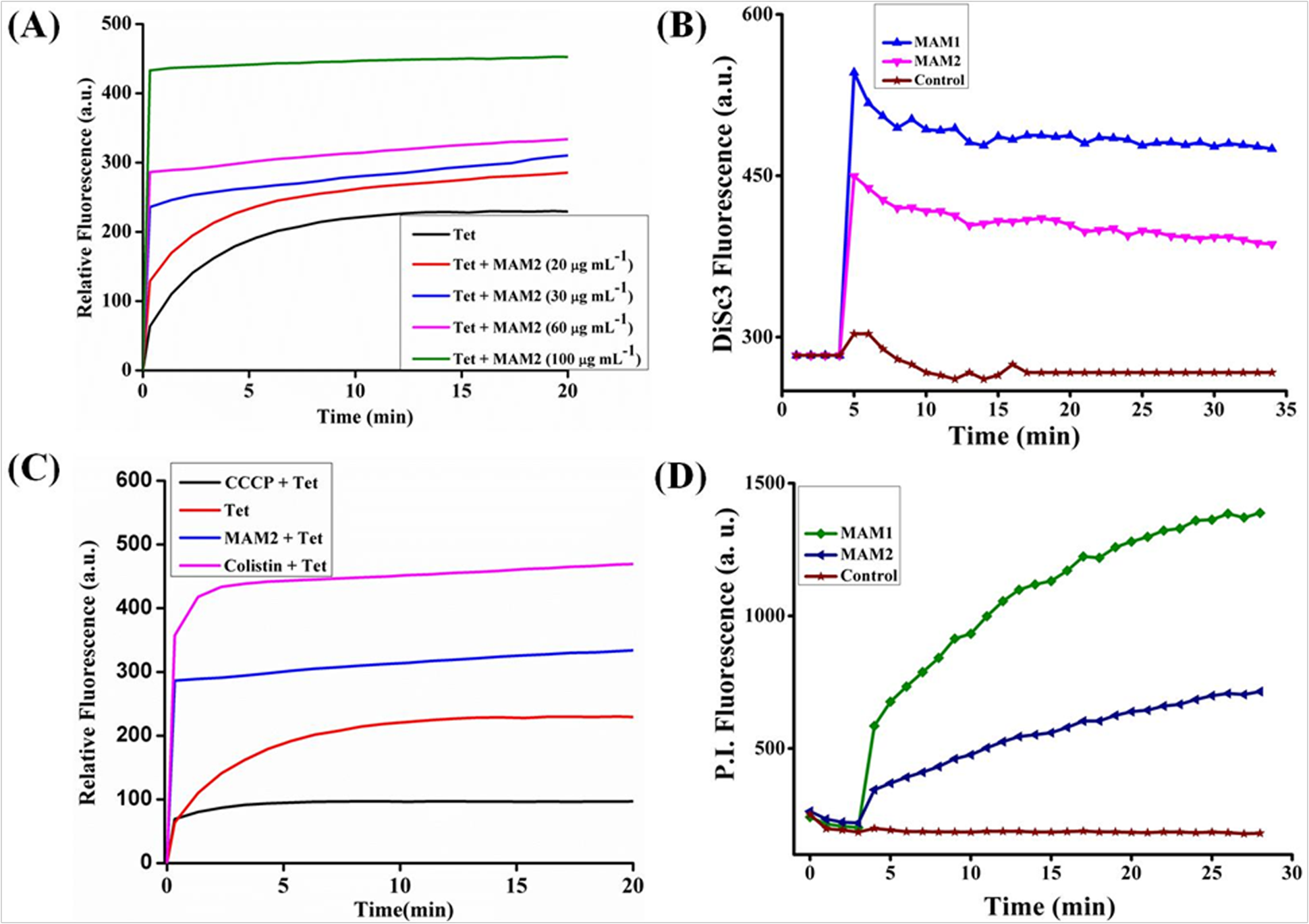
**

**Figure C. Mechanistic studies using tetracycline sensitive *E. coli* (ATCC 25922)*.*** (A) *E. coli* had much faster and higher uptake of tetracycline (Tet) in presence MAM2 (20, 30, 60 and 100 µg mL^-1^) than tetracycline alone (100 µg mL^-1^). (B) MAMs dissipated the membrane potential in *E. coli* as seen in the increase in fluorescence of DiSC_3_(5) after addition of MAMs at 25 µg mL^-1^; (C) Colistin and MAM2 (both at 60 µg mL^-1^) increased whereas carbonyl cyanide *m*-chlorophenyl hydrazone, CCCP (5 µg mL^-1^) decreased the uptake of tetracycline (100 µg mL^-1^). (D) MAMs caused cytoplasmic membrane permeabilization as seen in the increase in fluorescence of propidium iodide (PI) after addition of MAMs at 25 µg mL^-1^; Relative fluorescence was calculated by subtracting the fluorescence without the bacteria from the fluorescence of bacteria containing samples.


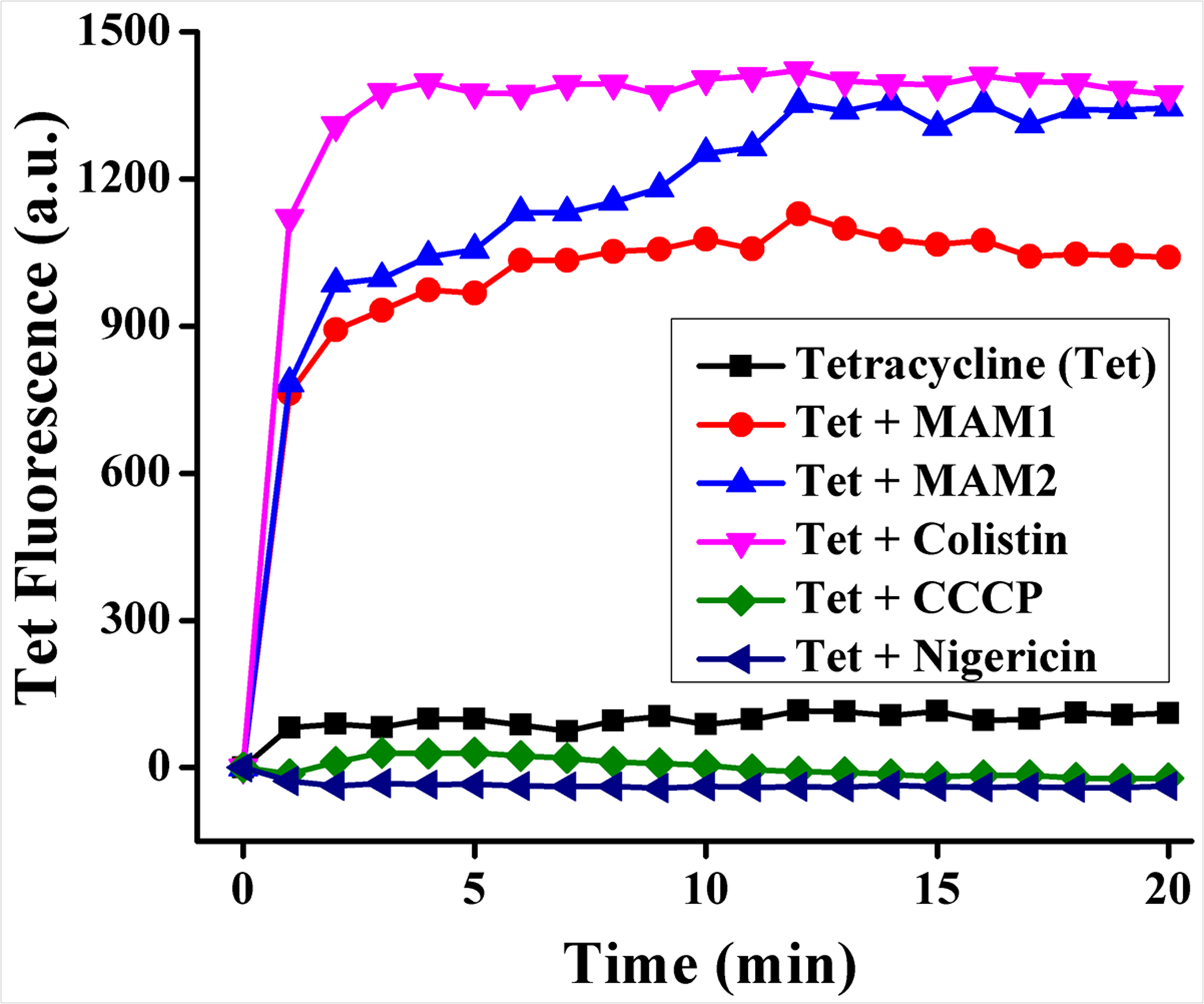


**Figure D. Uptake of tetracycline against *bla*_NDM-1_ *E. coli* R3336.** (A) Uptake of tetracycline (100 µg mL^-1^) in presence of the MAM1 and MAM2 (both at 20 µg mL^-1^) is much higher and faster compared to tetracycline alone as well as in the presence of nigericin and CCCP (both at 10 µg mL^-1^).

**Table A.** Antibacterial activity of conventional antibiotics in presence or absence of MAMs and colistin against MDR and *bla*_NDM-1_ clinical isolates

| **Antibiotics and**  **bacterial strains** |  | **MIC of antibiotic (µg mL^-1^)** | | | | |
| --- | --- | --- | --- | --- | --- | --- |
|  | **-MAMs** | **+MAM1**  (µg mL^-1^) | | **+MAM2**  (µg mL^-1^) | | **+Colistin**  (µg mL^-1^) |
|  |  | 12.5 | 25 | 25 | 50 | 0.25 |
| *bla_NDM-1_ E. coli R3336* |  |  |  |  |  |  |
| Ampicillin | >250 | >100 | >100 | >100 | >100 | >100 |
| Meropenem | 50 | 50 | 50 | 50 | 50 | 100 |
| Ciprofloxacin | 125 | >100 | >100 | 100 | 100 | 100 |
| Erythromycin | >250 | >100 | >100 | >100 | >100 | >100 |
| Kanamycin | >250 | >100 | >100 | >100 | >100 | >100 |
| *bla_NDM-1_ K. pneumoniae R3934* | | | | | | |
| Ampicillin | >250 | >100 | >100 | >100 | >100 | >100 |
| Meropenem | 50 | 50 | 50 | 50 | 25 | 100 |
| Ciprofloxacin | >250 | >100 | >100 | >100 | >100 | >100 |
| Erythromycin | >250 | >100 | >100 | >100 | >100 | 1.5 |
| Kanamycin | >250 | >100 | >100 | >100 | >100 | >100 |
| *bla_NDM-1_ K. pneumoniae ATCC-BAA-2146* | | | | | | |
| Ampicillin | >250 | ND | ND | ND | ND | ND |
| Meropenem | 25 | ND | ND | ND | ND | ND |
| Ciprofloxacin | >250 | ND | ND | ND | ND | ND |
| Erythromycin | 125 | ND | ND | ND | ND | ND |
| Kanamycin | >250 | ND | ND | ND | ND | ND |
| *MDR K. pneumoniae R3421* | | | | | | |
| Ampicillin | >250 | ND | ND | ND | ND | ND |
| Meropenem | 30 | ND | ND | ND | ND | ND |
| Ciprofloxacin | >250 | ND | ND | ND | ND | ND |
| Erythromycin | 125 | ND | ND | ND | ND | ND |
| Kanamycin | >250 | ND | ND | ND | ND | ND |

ND – Not determined

**Table B.** Antibacterial activity of MAMs and colistin against MDR and *bla*_NDM-1_ clinical isolates

| **Bacterial Strain MIC (µg mL^-1^)** | | | |
| --- | --- | --- | --- |
|  | MAM1 | MAM2 | Colistin |
| *bla_NDM-1_ E. coli R3336* | 250 | 125 | 0.75 |
| *bla_NDM-1_ K. pneumoniae R3934* | 125 | 125 | 1 |
| *bla_NDM-1_ K. pneumoniae ATCC-BAA-2146* | 62.5 | 125 | 0.75 |
| *MDR K. pneumoniae R3421* | 125 | 125 | 0.75 |

**Table C.** Synergistic profiles of tetracycline antibiotics in combination with MAMs or colistin against MDR and *bla*_NDM-1_ clinical isolates

| **Bacterial strains and FIC of MAMs** |  | **FIC^a^  FICI^b^** | | | | |
| --- | --- | --- | --- | --- | --- | --- |
| **Tetra Doxy Mino Tetra Doxy Mino** | | | | | | |
| *bla_NDM-1_ E. coli R3336* |  |  |  |  |  |  |
| FIC_MAM1_ = 0.05 | 0.4 | 0.1 | 0.4 | 0.45 | 0.15 | 0.45 |
| FIC_MAM1_ = 0.1 | 0.2 | 0.05 | 0.1 | 0.3 | 0.15 | 0.2 |
| FIC_MAM2_ = 0.2 | 0.8 | 0.2 | 0.2 | 1.0 | 0.4 | 0.4 |
| FIC_MAM2_ = 0.4 | 0.4 | 0.05 | 0.07 | 0.8 | 0.45 | 0.47 |
| FIC_Colistin_ = 0.3 | 0.8 | 0.4 | 0.8 | 1.1 | 0.7 | 1.1 |
| *bla_NDM-1_ K. pneumoniae R3934* | | | | | | |
| FIC_MAM1_ = 0.05 | 0.4 | 0.15 | 0.15 | 0.5 | 0.25 | 0.25 |
| FIC_MAM1_ = 0.1 | 0.2 | 0.04 | 0.02 | 0.4 | 0.24 | 0.22 |
| FIC_MAM2_ = 0.2 | 0.4 | 0.1 | 0.2 | 0.6 | 0.3 | 0.4 |
| FIC_MAM2_ = 0.4 | 0.4 | 0.04 | 0.03 | 0.8 | 0.44 | 0.43 |
| FIC_Colistin_ = 0.3 | 0.4 | 0.2 | 0.4 | 0.7 | 0.5 | 0.7 |
| *bla_NDM-1_ K. pneumoniae ATCC-BAA-2146* | | | | | | |
| FIC_MAM1_ = 0.05 | 0.02 | 0.01 | 0.1 | 0.22 | 0.21 | 0.3 |
| FIC_MAM1_ = 0.1 | 0.01 | 0.003 | 0.01 | 0.41 | 0.40 | 0.41 |
| FIC_MAM2_ = 0.2 | 0.05 | 0.003 | 0.01 | 0.25 | 0.20 | 0.21 |
| FIC_MAM2_ = 0.4 | 0.05 | 0.0008 | 0.003 | 0.45 | 0.40 | 0.40 |
| FIC_Colistin_ = 0.3 | 0.025 | 0.05 | 0.4 | 0.32 | 0.35 | 0.7 |
| *MDR K. pneumoniae R3421* | | | | | | |
| FIC_MAM1_ = 0.05 | 0.8 | 0.1 | 0.1 | 0.9 | 0.2 | 0.2 |
| FIC_MAM1_ = 0.1 | 0.14 | 0.03 | 0.04 | 0.34 | 0.23 | 0.24 |
| FIC_MAM2_ = 0.2 | 1.2 | 0.5 | 0.42 | 1.4 | 0.7 | 0.62 |
| FIC_MAM2_ = 0.4 | 0.2 | 0.08 | 0.08 | 0.6 | 0.48 | 0.48 |
| FIC_Colistin_ = 0.3 | 0.78 | 0.42 | 0.21 | 1.08 | 0.72 | 0.51 |

^a^Fractional Inhibitory Concentration (FIC) = [X]/MIC_X_, where [X] is the lowest inhibitory concentration of compound 1 in the presence of the compound 2. ^b^FIC index, FICI = FIC_compound1_ + FIC_compound2_. FICs were calculated only for the combination of tetracycline antibiotics and MAMs or colistin. Tetra, Doxy and Mino represent tetracycline, doxycycline and minocycline respectively.

**Table D. Effect of MAMs on the liver and kidney functional parameters and balance of electrolytes in the blood of mice 14 days post-treatment**

| Treatment | Liver Function | Kidney Function | | Electrolyte Balance | | |
| --- | --- | --- | --- | --- | --- | --- |
|  | ALP | Creatinine | Urea Nitrogen | Sodium ion | Potassium ion | Chloride |
|  | (IU L^-1^) | (mg dL^-1^) | (mg dL^-1^) | (mg dL^-1^) | (mg dL^-1^) | (mg dL^-1^) |
| PBS | 150 ± 57 | 0.23 ± 0.07 | 18.4 ± 3.4 | 143 ± 1.6 | 7.2 ± 0.7 | 111.5 ± 1.8 |
| MAM1 | 157.4 ± 59.8  (P > 0.05) | 0.14 ± 0.07  (P > 0.05) | 16.7 ± 3.1  (P < 0.05) | 137.5 ± 1.1  (P < 0.05) | 8.2 ± 1  (P < 0.05) | 105.3 ± 2.1  (P < 0.05) |
| MAM2 | 175 ± 44  (P > 0.05) | 0.13 ± 0.05  (P > 0.05) | 15 ± 2  (P > 0.05) | 139.6 ± 4.2  (P < 0.05) | 6.5 ± 0.5  (P < 0.05) | 106.8 ± 4.7  (P < 0.05) |
| Laboratory Range* | 209.3 ± 72.4 | 0.38 ± 0.12 | 16 ± 7.2 | 152.3 ± 17 | 8.9 ± 1.5 | 119.3 ± 13.5 |

The data are expressed as mean ± standard deviation, based on values obtained from 10 mice (n = 10). Statistical analysis was performed using student’s *t-test*. Differences are considered statistically significant with probability p < 0.05. ALP, alkaline phosphatase; I.U, international unit. *Source: Charles River Laboratories.
