# Supplementary material for: Correction: Membrane-Active Macromolecules Resensitize NDM-1 Gram-Negative Clinical Isolates to Tetracycline Antibiotics
Source: PLoS One. 2015 Apr 16;10(4):e0126757. doi: 10.1371/journal.pone.0126757 (PMC4400169; doi:10.1371/journal.pone.0126757)
Supplement: S1 File — (PDF) [file pone.0126757.s001.pdf]

RESEARCH ARTICLE

Membrane-Active Macromolecules Resensitize NDM-1 Gram-Negative Clinical Isolates to Tetracycline Antibiotics

Divakara S. S. M. Uppu[1], Goutham B. Manjunath[1], Venkateswarlu Yarlagadda[1], Jyothi E. Kaviyil[2], Raju Ravikumar[2], Krishnamoorthy Paramanandham[3], Bibek R. Shome[3], Jayanta Haldar[1]*

1 Chemical Biology & Medicinal Chemistry Laboratory, New Chemistry Unit, Jawaharlal Nehru Centre for Advanced Scientific Research (JNCASR), Jakkur, Bangalore, 560064, India, **2** Department of Neuromicrobiology, National Institute of Mental Health and Neuro Sciences (NIMHANS), Hosur Road, Bangalore, 560029, India, **3** National Institute of Veterinary Epidemiology and Disease Informatics (NIVEDI), Hebbal, Bengaluru, 560024, Karnataka, India

* jayanta@jncasr.ac.in

CrossMark
click for updates

OPEN ACCESS

Citation: Uppu DSSM, Manjunath GB, Yarlagadda V, Kaviyil JE, Ravikumar R, Paramanandham K, et al. (2015) Membrane-Active Macromolecules Resensitize NDM-1 Gram-Negative Clinical Isolates to Tetracycline Antibiotics. PLoS ONE 10(3): e0119422. doi:10.1371/journal.pone.0119422

Academic Editor: Chuanbing Tang, University of South Carolina, UNITED STATES

Received: November 3, 2014

Accepted: January 13, 2015

Published: March 19, 2015

Copyright: © 2015 Uppu et al. This is an open access article distributed under the terms of the Creative Commons Attribution License, which permits unrestricted use, distribution, and reproduction in any medium, provided the original author and source are credited.

Data Availability Statement: All relevant data are within the manuscript and its Supporting Information files.

Funding: This work was supported by the grant "Ramanujan fellowship" [SR/S2/RJN-43/2009] from the Department of Science and Technology (DST), Govt. of India. The funders had no role in study design, data collection and analysis, decision to publish, or preparation of the manuscript.

Competing Interests: Membrane-active molecules, discovered by DSSMU and JH, described in the

Abstract

Gram-negative 'superbugs' such as New Delhi metallo-beta-lactamase-1 (bla_{NDM-1}) producing pathogens have become world's major public health threats. Development of molecular strategies that can rehabilitate the 'old antibiotics' and halt the antibiotic resistance is a promising approach to target them. We report membrane-active macromolecules (MAMs) that restore the antibacterial efficacy (enhancement by >80-1250 fold) of tetracycline antibiotics towards bla_{NDM-1} *Klebsiella pneumonia* and bla_{NDM-1} *Escherichia coli* clinical isolates. Organismic studies showed that bacteria had an increased and faster uptake of tetracycline in the presence of MAMs which is attributed to the mechanism of re-sensitization. Moreover, bacteria did not develop resistance to MAMs and MAMs stalled the development of bacterial resistance to tetracycline. MAMs displayed membrane-active properties such as dissipation of membrane potential and membrane-permeabilization that enabled higher uptake of tetracycline in bacteria. *In-vivo* toxicity studies displayed good safety profiles and preliminary *in-vivo* antibacterial efficacy studies showed that mice treated with MAMs in combination with antibiotics had significantly decreased bacterial burden compared to the untreated mice. This report of re-instating the efficacy of the antibiotics towards bla_{NDM-1} pathogens using membrane-active molecules advocates their potential for synergistic co-delivery of antibiotics to combat Gram-negative superbugs.

Introduction

The WHO Global Report on Surveillance of Antimicrobial Resistance 2014 says that *E. coli* and *K. pneumoniae* have developed more than 50% of resistance to commonly used antibacterial drugs in many settings [1]. Of particular concern is the fact that *K. pneumoniae* has developed resistance to carbapenems, the last line of available treatment in all WHO regions [1].

manuscript have been filed for a PCT application (WO 2014006601 A3). The authors' confirm that this does not alter their adherence to all the PLOS ONE policies on sharing data and materials.

This ongoing explosion of multi-drug resistant Gram-negative 'superbugs' including the New Delhi metallo-beta-lactamase-1 (bla_{NDM-1}) producing bacteria along with the paucity of antibiotics poses a threat to the global public health [2–4]. More importantly, carbepenem resistant bacteria such as bla_{NDM-1} *E. coli* and bla_{NDM-1} *K. pneumoniae* have become resistant to the highly toxic and the last resort antibiotic, colistin [2,3]. Another antibiotic, tigecycline though useful in tissue infections, is less useful in systemic infections. Development of resistance to tigecycline in Gram-negative bacteria being reported in clinical settings is a matter of worry [4]. The emergence of pan-drug resistant Gram-negative bacteria has become a reality, necessitating the exploration for alternative ammunitions to combat them.

One of the promising strategies to target multi-drug resistant (MDR) bacteria is by using either combination of two antibiotics or combination of antibiotics and non-antibiotics/adjuvants [5–16]. The combination of two or more antibiotics has been found to foster the development of bacterial resistance than the individual antibiotics [17]. An ideal combination approach would have at least one agent with lower rate of emergence of bacterial resistance. Molecules targeting the cell membrane of bacteria have been known to possess low propensity for triggering the development of bacterial resistance [18–24]. We have recently reported quaternized poly (isobutylene-alt-*N*-(*N*',*N*'-dimethyl *N*'-alkyl aminopropyl)-maleimide) derivatives with membrane-active properties having excellent antibacterial efficacy against a panel of drug-resistant bacteria [23]. Thus, it would be ideal to entrust these polymeric derivatives, designated herein as membrane-active macromolecules (MAMs) (Fig. 1A), the role of re-sensitizing the superbugs to the antibiotics using a combination approach. In this report, we took an approach of developing molecular strategies to rehabilitate the 'old antibiotics' towards Gram-negative superbugs and attempted to support our observations using organismic studies and molecular reasoning, bridging both chemistry and biology. The mechanistic investigations of re-sensitization, membrane-active properties of MAMs and more importantly, the drug resistance studies of tetracycline antibiotics in presence of MAMs are reported herein.

Results and Discussion

Clinical isolates of carbapenem-resistant bacteria (minimum inhibitory concentration, MIC of meropenem >16 μg mL^{-1}) were characterized for bla_{NDM-1} gene (475 bp) using PCR and gel electrophoresis (Fig. 1B) [2,25,26] .The bla_{NDM-1} gene was confirmed in *K. pneumoniae* R3934 and *E. coli* R3336 including the positive control *K. pneumoniae* (ATCC-BAA-2146) whereas MDR *K. pneumoniae* R3421 showed negative (Fig. 1B). The prevalence of bla_{NDM-1} in clinical settings makes the development of alternative strategies imperative.

All the three bla_{NDM-1} isolates and the MDR strain are represented here on as R3336, R3934, ATCC2146 and R3421 respectively. All the four isolates were sensitive to tigecycline and colistin only (MIC = 0.5–1 μg mL^{-1}) and were highly resistant to intracellular antibiotics (ciprofloxacin, kanamycin, erythromycin, tetracycline, doxycycline and minocycline) and cell-wall directed antibiotics (ampicillin and meropenem) (see Table A in S1 Text) [25]. In most of the cases, the MICs of the antibiotics against all the four bacteria were > 250 μg mL^{-1}, the highest concentration tested (see Table A in S1 Text). This provides yet another evidence for the high level of multi-drug resistance in clinical settings. The antibacterial activity of MAMs alone was also evaluated and found to be marginal against all the four isolates. MAM1 displayed MIC of 250 μg mL^{-1} against R3336 and 62.5 μg mL^{-1} against ATCC2146 whereas it showed MIC of 125 μg mL^{-1} against both R3934 and R3421 (see Table B in S1 Text). On the other hand, MAM2 showed MIC of 125 μg mL^{-1} against all the four bacteria (see Table B in S1 Text).

We tested the combination efficacy of eight antibiotics and MAMs against the isolates (see Table A in S1 Text) using chequerboard assays [27]. Using the fractional inhibitory

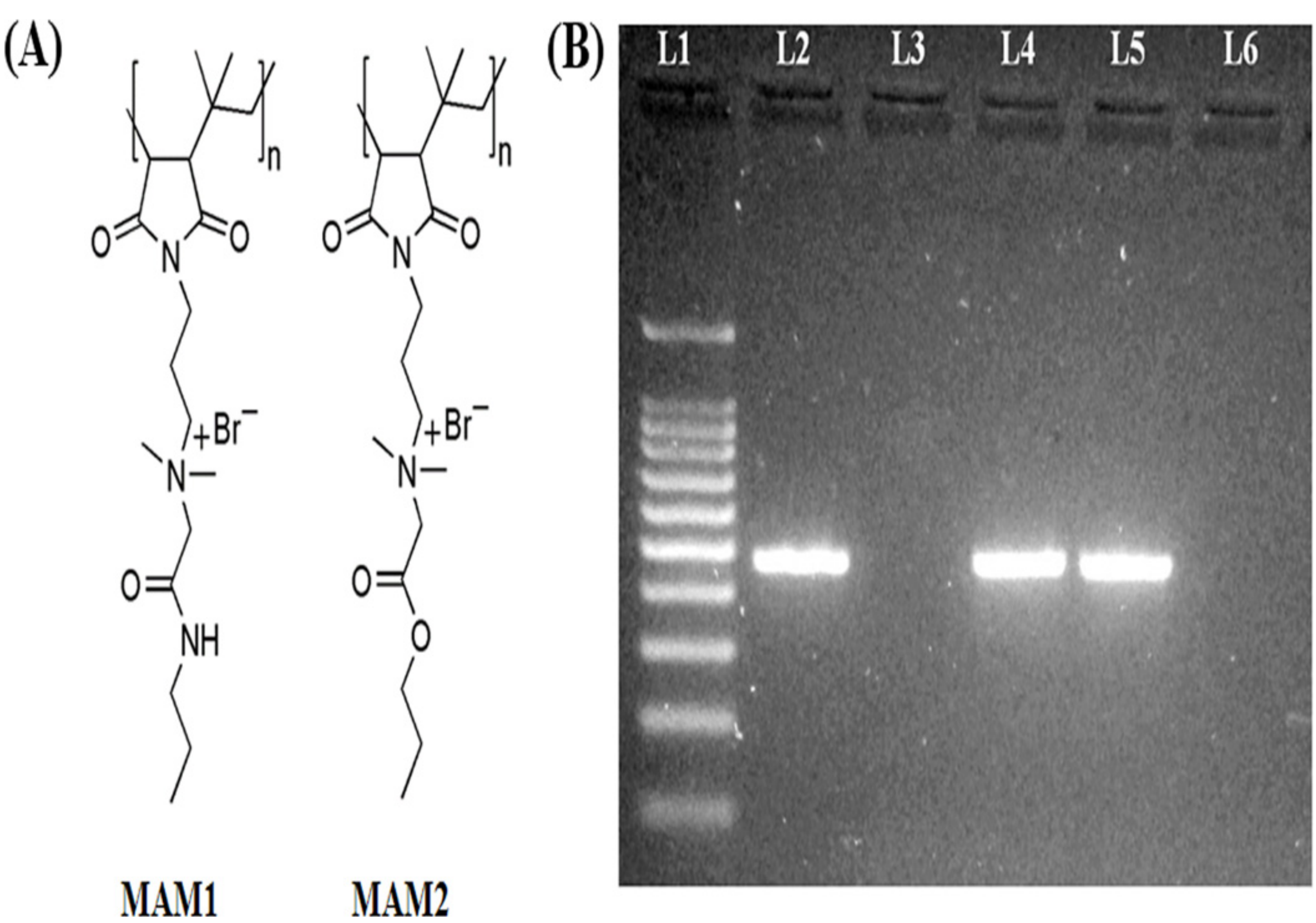

Fig 1. (A) Structures of the membrane active molecules (MAMs) and (B) Schematic representation of agarose gel (2%) showing the 475 bp amplified product by conventional polymerase chain reaction. Lane 1, 100 bp DNA ladder; Lane 2, positive control- NDM-1 producing *K. pneumoniae* (ATCC-BAA-2146); Lane 3, negative control- *E. coli* (ATCC-25922); Lane 4, *E. coli* R3336 and Lane 5, *K. pneumoniae* R3934 confirm the $bla_{\text{NDM-1}}$ gene; Lane 6, multi-drug resistant (MDR) *K. pneumoniae* R3421 which was negative for $bla_{\text{NDM-1}}$ gene.

doi:10.1371/journal.pone.0119422.g001

concentration (FIC), a combination was called synergistic when the combined FIC of both agents, (the FIC Index; FICI) was ≤ 0.5 [27]. In the presence of MAMs (at a concentration of 12.5 and 25 μg mL^{-1} for MAM1 and 25 and 50 μg mL^{-1} for MAM2), the MICs of the antibiotics like ampicillin, meropenem, ciprofloxacin, erythromycin and kanamycin did not reduce when tested against the two clinical isolates- R3336 and R3934 (see Table A in S1 Text). However, synergistic effect (FICI of ≤ 0.5) of MAMs was observed with tetracycline antibiotics (tetracycline, doxycycline and minocycline) against all the four isolates (see Table C in S1 Text).

Notably, MAM1 at 12.5 μg mL^{-1}, which is 1/20th, 1/10th and 1/5th of its MIC against R3336, R3934 and R3421 respectively showed high synergy (FICI as low as 0.15) in combination with both doxycycline and minocycline (see Table C in S1 Text). At 25 μg mL^{-1}, MAM1 in combination with doxycycline and minocycline displayed synergy against all the four isolates. On the other hand, MAM2 at 25 μg mL^{-1} (1/5th of MIC) resulted in synergistic combinations with doxycycline and minocycline against the $bla_{\text{NDM-1}}$ strains (ATCC2146, R3336 and R3934). Whereas, at 50 μg mL^{-1}, it showed synergy with doxycycline and minocycline against all the four clinical isolates. Tetracycline showed synergy in presence of MAM1 against R3336, R3934 and ATCC 2146 whereas in presence of MAM2, synergy was observed only against

ATCC2146. Next, we tested the combination efficacy of eight antibiotics and colistin against all the four clinical isolates as a control. Colistin at 0.25 μg mL^{-1} (~ $1/3^{rd}$ of MIC) displayed synergy (FICI of 0.3 to 0.5) with tetracycline against R3934 and showed synergy with both tetracycline and doxycycline against ATCC2146 (see Table C in S1 Text).

The study also demonstrated that MAMs re-sensitized all the four isolates to tetracycline antibiotics (Table 1). The CLSI breakpoint for the susceptibility of tetracycline antibiotics is $\leq$ 4 μg mL^{-1} [24]. The MICs of tetracycline antibiotics were brought down to susceptible limits of as low as 0.2–3.1 μg mL^{-1}, resulting in >80–1250 fold of reduction in the presence of MAMs (Table 1). MAM1 at 12.5 μg mL^{-1} ($1/10^{th}$ of its MIC) reduced the MIC of doxycycline and minocycline (~10 fold) to 3.1 μg mL^{-1} against R3421 (Table 1). Against ATCC 2146, MAM1 reduced the MIC of doxycycline (> 80 fold) to 3.1 μg mL^{-1} at $1/5^{th}$ of its MIC (12.5 μg mL^{-1}). Whereas, at 25 μg mL^{-1}, MAM1 re-sensitized all the four clinical isolates to tetracycline antibiotics (MIC = 0.8–3.1 μg mL^{-1}) with the reduction in the range of > 80–312 fold. On the other hand, MAM2 at 25 μg mL^{-1} ($1/5^{th}$ of MIC) decreased the MIC of doxycycline (> 312 fold) and minocycline (~ 77 fold) down to 0.8 μg mL^{-1} against ATCC2146. At 50 μg mL^{-1}, MAM2 re-sensitized (> 300–1250 fold) all the four isolates to tetracycline antibiotics by lowering the MIC to 0.2–3.1 μg mL^{-1}. Over all, MAM1 showed higher synergistic and re-sensitization profiles at low concentrations than MAM2 against all the four isolates. The synergistic combination was not only found to be bacteriostatic but also bactericidal, unlike tetracycline antibiotics that are only bacteriostatic in nature. MAM1 + minocycline (50 μg mL^{-1} + 6.3 μg mL^{-1}) showed bactericidal activity whereas minocycline (6.3 μg mL^{-1}) alone and MAM1 (50 μg mL^{-1}) alone were devoid of antibacterial activity against R3336 (Fig. 2A and see Figure A in S1 Text). Colistin, at 0.25 μg mL^{-1} showed no re-sensitization of any of the four isolates to tetracycline antibiotics (Table 1). MAMs had higher synergistic and re-sensitization profiles than colistin. The synergistic and re-sensitization profiles of MAMs and colistin are seen specifically to tetracycline antibiotics but not with other cell-wall directed or intracellular antibiotics. In presence of either MAMs or colistin, synergy was not observed with ampicillin and meropenem as the beta-lactamases found in the MDR and $bla_{\mathrm{NDM-1}}$ Gram-negative bacteria degrade them [28]. Within tetracycline antibiotics, it was found that the synergistic and re-sensitization profiles were always way better for doxycycline and minocycline than the first generation antibiotic, tetracycline. Over all, four isolates of Gram-negative superbugs were made susceptible to tetracycline antibiotics in combination with MAMs. Even against a tetracycline sensitive strain of *E. coli* (ATCC 25922), MAM2 at 62.5 μg mL^{-1} ($1/4^{th}$ of its MIC) showed a 2-fold reduction in the MIC of tetracycline (MIC-1.56 μg mL^{-1}).

Emergence of rapid resistance in bacteria to conventional antibiotics is a major problem and is the primary hurdle for the introduction of new antibiotics in clinical settings. We have evaluated the development of resistance in $bla_{\mathrm{NDM-1}}$ *E. coli* (R3336) by exposing to sub-MIC doses of colistin, MAM1, minocycline alone and in combination with MAM1 over 30 passages (Fig. 2B). As shown in Fig. 2B, $bla_{\mathrm{NDM-1}}$ *E. coli* (R3336) rapidly developed resistance to colistin with an increase in MIC (from 0.5 μg mL^{-1} on day 1) up to 1000 μg mL^{-1} within 15 passages. This supports the high emergence of resistance in Gram-negative bacteria to colistin that is being observed in clinical settings [3]. In contrast, resistance did not develop with the treatment of MAM1 as observed by the consistent MIC over 30 passages as shown in the Fig. 2B. This data shows that bacteria do not readily develop resistance to membrane-active molecules. $Bla_{\mathrm{NDM-1}}$ *E. coli* (R3336) which was already resistant to minocycline further developed resistance by a 16-fold increase in MIC after 30 passages (Fig. 2B). Once again, it suggests that resistance to tetracycline antibiotics can indeed develop rapidly with sub-MIC treatment. On the other hand, the development of resistance by $bla_{\mathrm{NDM-1}}$ *E. coli* (R3336) to minocycline was not observed in the presence of MAMs (Fig. 2B). The MIC of MAM1 + minocycline (50 μg mL^{-1} + 6.2 μg mL^{-1}) stayed consistent over

Table 1. Antibacterial efficacy of tetracycline antibiotics with or without MAMs and colistin against MDR and bla_{NDM-1} clinical isolates.

Antibiotics and bacterial strains	MIC of antibiotic (μg mL^{-1})					
	-MAMs	+MAM1 (μg mL^{-1})		+MAM2 (μg mL^{-1})		+Colistin (μg mL^{-1})
		12.5	25	25	50	0.25
blaNDM-1 E. coli R3336						
Tetracycline	32	12.5	6.2	25	12.5	25
Doxycyline	62	6.2	3.1	12.5	3.1	25
Minocycline	32	12.5	3.1	6.2	2.2	25
blaNDM-1 K. pneumoniae R3934						
Tetracycline	>250	100	50	>100	100	100
Doxycycline	>250	37	9.4	25	9.4	50
Minocycline	62	9.4	1.5	12.5	1.9	25
blaNDM-1 K. pneumoniae ATCC-BAA-2146						
Tetracycline	>250	6.2	3.1	12.5	12.5	6.2
Doxycycline	>250	3.1	0.8	0.8	0.2	12.5
Minocycline	62	6.2	0.8	0.8	0.2	25
MDR K. pneumoniae R3421						
Tetracycline	16	12.5	2.3	19	3.1	12.5
Doxycycline	30	3.1	0.8	16	2.3	12.5
Minocycline	30	3.1	1.1	12.5	2.3	6.2

doi:10.1371/journal.pone.0119422.t001

the 30 passages as shown in Fig. 2B. Resistance studies performed in tetracycline sensitive *E. coli* (ATCC 2592) also showed similar results wherein the resistance did not develop for the combination of tetracycline and MAMs unlike tetracycline alone (see Figure B in S1 Text). This showed that *E. coli* did not develop resistance to tetracycline antibiotics in presence of MAMs. It is known that the membrane-active nature bestows low tendency to develop bacterial resistance to membrane-lytic agents [18–24]. We believe that it is the same membrane-active properties that stalled the antibiotic resistance of tetracycline but it certainly needs further investigations. These observations prove the advantage of using MAMs in combination with antibiotics to curb resistance development.

In-vivo toxicity of MAMs still remain unaddressed that prevent them to be suitable candidates for therapeutic applications. Experiments were performed to assess the *in-vivo* systemic toxicity of MAMs after single-dose intravenous (i.v.) administration to mice. Mice were observed for 14 days post treatment to find out the lethal effects. The 50% lethal dose (LD_{50}) values were found to be 20 mg kg^{-1} and 37 mg kg^{-1} for MAM1 and MAM2 respectively. We also tested the toxicity of the MAM1 through single dose intraperitoneal (i. p.) and subcutaneous (s. c.) injection into the mice and found no lethal effects up to 17.5 mg kg^{-1} and 55 mg kg^{-1} respectively. Next, we investigated the sub-chronic toxicity to major organs in mice by evaluating the clinical biochemistry parameters in the blood after a single-dose i.v. administration of MAM1 and MAM2 (at a dosage of 2 mg kg^{-1}). Neither of the derivatives induced any adverse toxicity to major organs like liver and kidney and did not interfere with the balance of electrolytes in the blood of mice 2 days (Table 2) and 14 days (see Table D in S1 Text) post treatment compared to vehicle control and laboratory parameters. For the combination efficacy of antibiotics and MAMs, the mice were given a single i.v. injection of combination of doxycycline and MAM1. It was observed that the mice showed no lethal effects after injection of doxycycline + MAM1 up to 100 mg kg^{-1} + 15 mg kg^{-1} and also doxycycline (100 mg kg^{-1}) alone. This showed that these

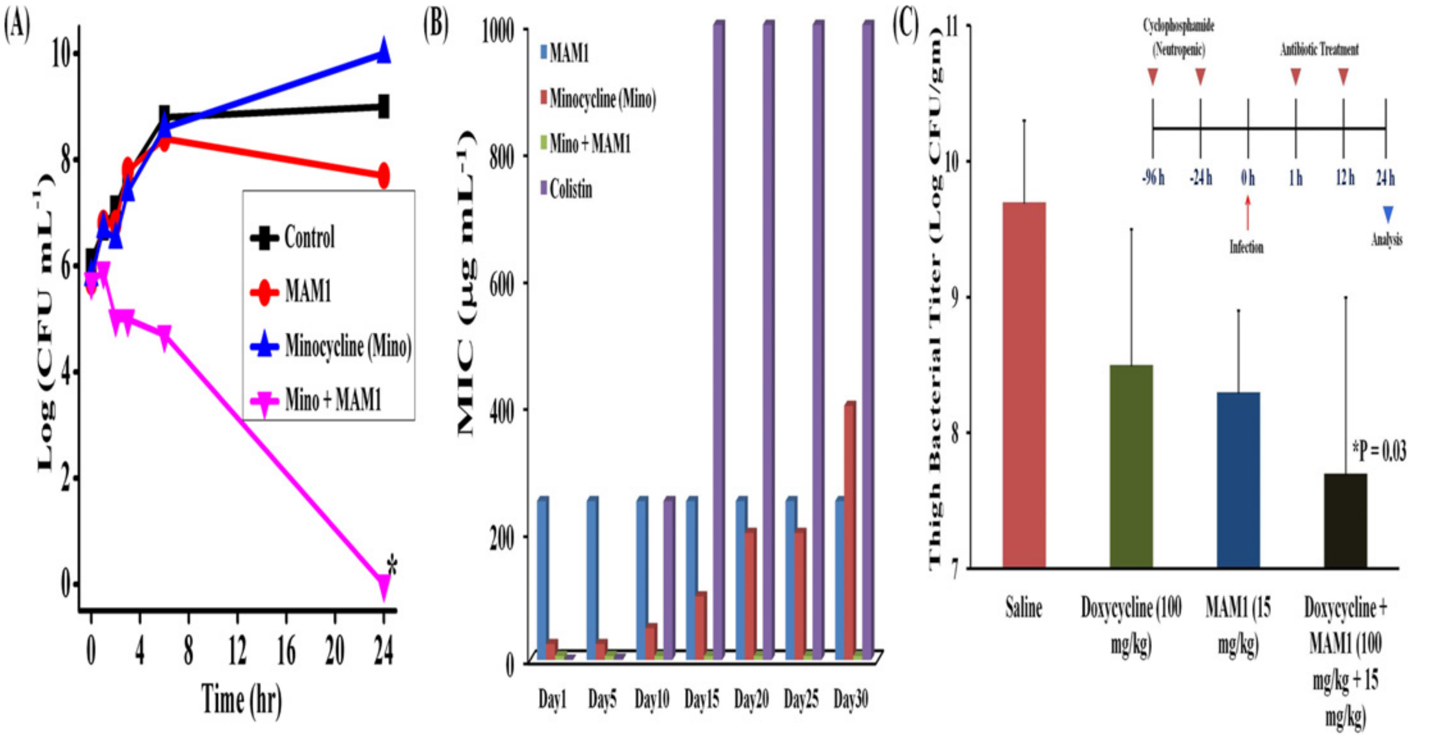

Fig 2. Synergy, resistance development and *in-vivo* antibacterial efficacy of MAMs and tetracycline antibiotics against bla_{NDM-1} *E. coli* (R3336). (A) The combination of MAM1 and minocycline (50 μg mL^{-1} + 6.3 μg mL^{-1}) showed synergistic bactericidal activity whereas MAM1 (50 μg mL^{-1}) alone and minocycline alone (6.3 μg mL^{-1}) were devoid of antibacterial activity (star represents < 50 CFU mL^{-1}, the detection limit of the experiment). (B) Bacteria developed resistance to minocycline alone with increase in MIC up to 400 μg mL^{-1} whereas it did not develop resistance to minocycline (6.2 μg mL^{-1}) in presence of MAM1 (50 μg mL^{-1}) up to 30 days. Bacteria did not readily develop resistance to MAM1 whereas it developed resistance to colistin with an increase in MIC up to 1000 μg mL^{-1}. (C) *In-vivo* antibacterial efficacy of MAM1 and doxycycline in mice models. The bacterial burden in the thighs of the mice (4 mice in each group) were determined and expressed as mean ± S.E.M (standard error of mean). P value was calculated using the unpaired Student's *t* test (2 tailed 2 samples assuming equal variances) and a value $P < 0.05$ was considered significant. *P = 0.03 between the saline treated and combination treated samples (Inset shows the experimental design).

doi:10.1371/journal.pone.0119422.g002

Table 2. Effect of MAMs on the liver and kidney functional parameters and balance of electrolytes in the blood of mice 48 h post-treatment.

Treatment	Liver Function	Kidney Function		Electrolyte Balance		
	ALP (IU L^{-1})	Creatinine (mg dL^{-1})	Urea Nitrogen (mg dL^{-1})	Sodium ion (mg dL^{-1})	Potassium ion (mg dL^{-1})	Chloride (mg dL^{-1})
PBS	150 ± 57	0.23 ± 0.07	18.4 ± 3.4	143 ± 1.6	7.2 ± 0.7	111.5 ± 1.8
MAM1	157 ± 64 (P > 0.05)	0.19 ± 0.06 (P > 0.05)	16.4 ± 2.2 (P > 0.05)	143 ± 1.7 (P > 0.05)	7.3 ± 0.5 (P > 0.05)	109 ± 2.4 (P < 0.05)
MAM2	140 ± 43 (P > 0.05)	0.25 ± 0.11 (P > 0.05)	19.1 ± 4.4 (P > 0.05)	146.1 ± 0.8 (P < 0.05)	6.0 ± 0.5 (P < 0.05)	114 ± 2.1 (P < 0.05)
Laboratory Range*	209.3 ± 72.4	0.38 ± 0.12	16 ± 7.2	152.3 ± 17	8.9 ± 1.5	119.3 ± 13.5

The data are expressed as mean ± standard deviation, based on values obtained from 10 mice (n = 10). Statistical analysis was performed using student's *t-test*. Differences are considered statistically significant with probability $p < 0.05$. ALP, alkaline phosphatase; I.U, international unit.
*Source: Charles River Laboratories.

doi:10.1371/journal.pone.0119422.t002

MAMs alone and in combination with antibiotics have low toxicity in mice models and have a good safety profile required for therapeutic applications.

Preliminary studies were performed to evaluate the *in-vivo* antibacterial efficacy of combination of MAMs and antibiotics. Using a neutropenic thigh infection model against $bla_{\mathrm{NDM-1}}$ *E. coli* (R3336), mice were injected (1 h and 12 h post-infection) with MAM1 (15 mg kg^{-1}), doxycycline (100 mg kg^{-1}) and MAM1 + doxycyline (15 mg kg^{-1} + 100 mg kg^{-1}). The bacterial burdens in thigh muscle of the mice treated with the combination of MAM 1 + doxycyline were found to be significantly (P = 0.03) lower than the saline treated mice (Fig. 2C). These preliminary studies though show some potential of combination therapy but certainly needs further investigations.

Mechanistic studies were performed in tetracycline resistant $bla_{\mathrm{NDM-1}}$ *E. coli* (R3336) and tetracycline sensitive *E. coli* (ATCC 25922) to understand the mechanism of re-sensitization of tetracycline antibiotics. Tetracycline is fluorescent in aqueous solutions and it is reported that there is an enhancement in its fluorescence as it traverses the bacterial cell membranes [29]. The uptake of tetracycline (100 μg mL^{-1}) alone was less and slow in $bla_{\mathrm{NDM-1}}$ *E. coli* (R3336) as shown in Fig. 3A. In presence of the MAMs (20 μg mL^{-1}), $bla_{\mathrm{NDM-1}}$ *E. coli* (R3336) had a much higher and faster uptake of tetracycline compared to tetracycline alone (Fig. 3A). Similarly, the higher uptake was observed even with tetracycline sensitive *E. coli* (ATCC 25922) (see Figure C in S1 Text). The mechanism of re-sensitization can be attributed to this enhanced and faster uptake of tetracycline.

In a pursuit to determine the driving force for this higher uptake of tetracycline, we investigated the membrane-active properties of MAMs. The urge to do so stems from the fact that tetracycline is known to accumulate in bacteria through ΔpH (transmembrane pH) gradient [30]. Bacteria maintain an intact proton motive force (PMF) across the cell membrane that is a combination of transmembrane electrical potential (ΔΨ) and ΔpH [31]. Using a fluorescence assay, we measured the cytoplasmic membrane depolarization by MAMs in $bla_{\mathrm{NDM-1}}$ *E. coli* (R3336) using the dye, $DiSC_3(5)$ (3, 3'-dipropylthiadicarbocyanine iodide) (Fig. 3B). $DiSC_3(5)$ intercalates into the cytoplasmic membrane of energized cells and initially the fluorescence is quenched [23,24]. Upon disruption of the membrane potential (ΔΨ), the release of the dye into the solution results in increase of fluorescence. MAMs selectively dissipated the ΔΨ component of the PMF as seen in the increase of fluorescence of $DiSC_3(5)$ when added at 100 μg mL^{-1} (Fig. 3B). MAM1 showed higher cytoplasmic membrane depolarization than MAM2 (Fig. 3B). This observation supports the fact that MAM1 displayed higher synergy and re-sensitization profiles than MAM2 (Table 1 and see Table C in S1 Text). Colistin, like MAMs, is already known to dissipate ΔΨ [6] whereas tetracycline alone did not have any effect on the ΔΨ of bacteria (Fig. 3B). Even against tetracycline sensitive *E. coli* (ATCC 25922), MAM1 has higher dissipation of membrane potential than MAM2 (see Figure C in S1 Text). This selective dissipation of ΔΨ by MAMs led us to the hypothesis that membrane potential might play a role in the mechanism of re-sensitization.

To support this hypothesis, we performed the uptake studies of tetracycline in $bla_{\mathrm{NDM-1}}$ *E. coli* (R3336) in presence of CCCP (carbonyl cyanide *m*-chlorophenyl hydrazone) that is known to abolish PMF and nigericin which is known to selectively dissipate ΔpH [30,31]. Bacteria in presence of both nigericin and CCCP (both at 10 μg mL^{-1}) had a decreased uptake of tetracycline (Fig. 3C and see Figure D in S1 Text). Whereas, in presence of MAMs (both at 20 μg mL^{-1}) and colistin (30 μg mL^{-1}), increased uptake of tetracycline was observed compared to tetracycline alone (Fig. 3C). Even tetracycline sensitive *E. coli* (ATCC 25922) had a lower uptake in presence of CCCP whereas colistin and MAMs had an increased uptake of tetracycline (see Figure C in S1 Text). As tetracycline is known to accumulate in the bacteria through ΔpH dependent pathway [30], CCCP and nigericin resulted in a decreased uptake of tetracycline.

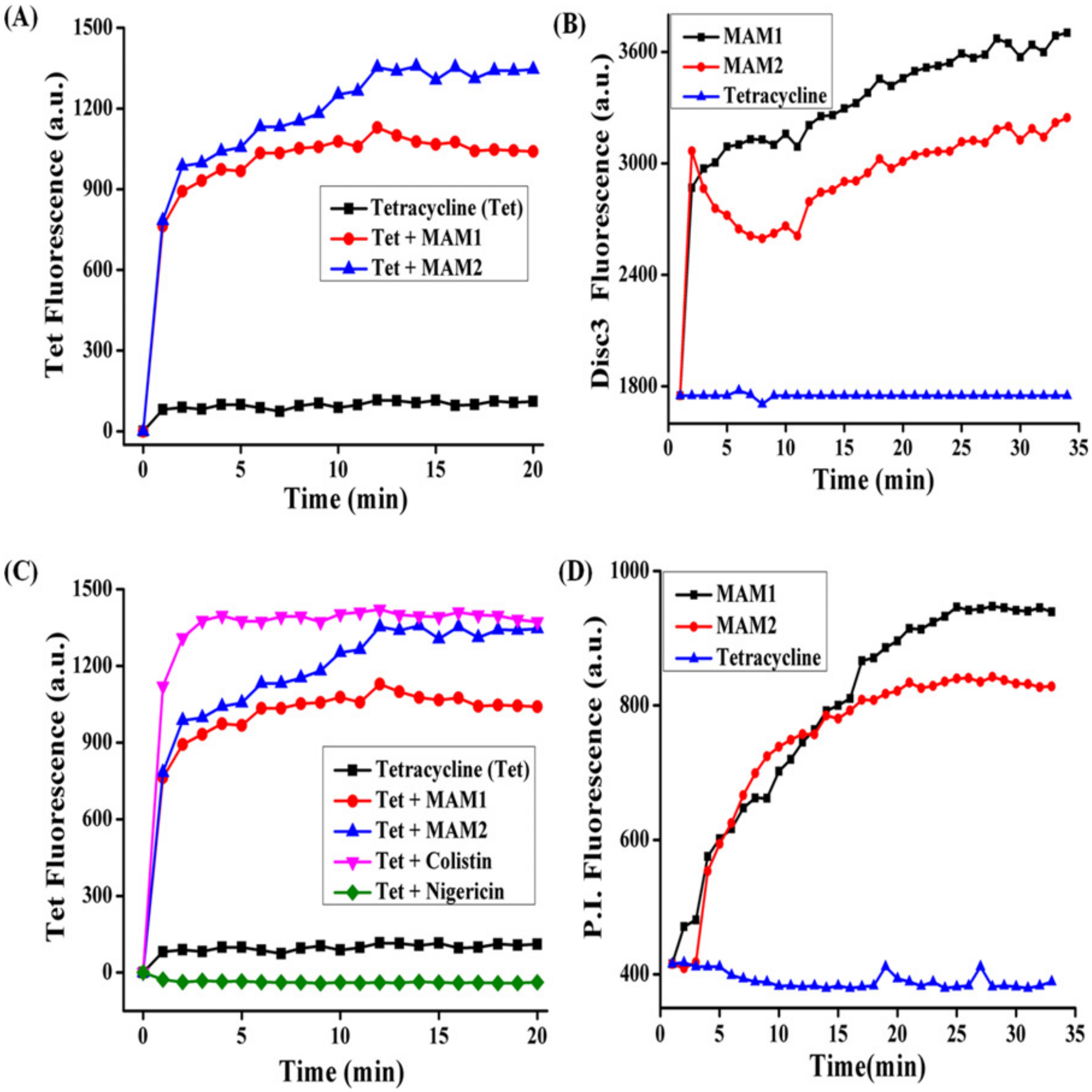

Fig 3. Uptake of tetracycline and membrane-active properties of polymers against *bla*$_{\text{NDM-1}}$ *E. coli* (R3336). (A) Uptake of tetracycline by increase in its fluorescence in presence of the MAM1 and MAM2 ((both at 20 μg mL^{-1}). Tetracycline was used at 100 μg mL^{-1}. (B) Dissipation of membrane potential by increase in the fluorescence of DiSC$_3$(5) with the treatment of MAM1 and MAM2 (both at 100 μg mL^{-1}). (C) Uptake of tetracycline in presence of MAM1, MAM2 (both at 20 μg mL^{-1}) and colistin (30 μg mL^{-1}) as well as in the presence of nigericin (at 10 μg mL^{-1}). (D) Membrane permeabilization by increase in the fluorescence of propidium iodide (PI) with the treatment of MAM1 and MAM2 (both at 100 μg mL^{-1}).

doi:10.1371/journal.pone.0119422.g003

However, MAMs dissipate the ΔΨ component of the PMF that might have to be compensated by an increase in the ΔpH component driving the uptake of tetracycline. It has been reported that CCCP and nigericin inhibits tetracycline accumulation whereas valinomycin, which specifically cancels the ΔΨ, does not inhibit the accumulation [30].

Kinetics of membrane permeabilization was studied by measuring the uptake of a fluorescent probe propidium iodide (PI) against *bla*$_{\text{NDM-1}}$ *E. coli* (R3336). This dye enters only membrane compromised cells and fluoresces upon binding to nucleic acids [23,24]. MAMs when added at 100 μg mL^{-1}, increased the permeability of propidium iodide (PI) to bacterial cell membranes as seen by the increase of its fluorescence whereas tetracycline alone does not have

any effect on membrane permeability (Fig. 3D). MAM1 caused higher cytoplasmic membrane permeabilization than MAM2 which further facilitated the uptake of tetracycline antibiotics (Fig. 3D). Similar results were also observed with tetracycline sensitive *E. coli* (ATCC 25922) (see Figure C in S1 Text). Overall, the mechanism of restoring the efficacy can be attributed to the membrane-active properties of MAMs that enabled the uptake of tetracycline both in tetracycline resistant and sensitive strains.

Clinically approved drugs (loperamide) [6], 2-aminoimidazoles [7], acyl lysine oligomers (OAKs) [9], polycarbonates [10], silver (Ag^+) [14], Ianthelliformisamines [15] and cationic cobaltocenium-containing polymers [16] were found to be antibiotic-enhancers against bacteria. These reports taken together with our data, suggest that the synergy to different antibiotics mostly depends on the nature of the membrane-active agent used in combination to them. Our observations and mechanistic studies suggest that the synergy is indeed selective to tetracycline antibiotics but not to other intracellular antibiotics like macrolides, fluoroquinolones and aminoglycosides. To a large extent, bacteria develop resistance to tetracycline antibiotics by excluding them through RND-efflux pumps whereas, other intracellular antibiotics have additional major mechanisms of resistance such as target modification and inactivating enzymes [28]. So, we believe that it is the membrane-active properties of MAMs that increased the uptake of tetracycline antibiotics giving them a backdoor entry into the bacteria that are otherwise excluded through the efflux pumps. However, as observed by our group and others, since MAMs dissipate the $\Delta\Psi$, the uptake of aminoglycosides is not favorable as they need the $\Delta\Psi$ for their uptake [6]. Despite the fact that membrane-active properties of MAMs can facilitate the entry of other intracellular antibiotics, target modification and inactivating enzymes can play a major role in making them ineffective.

Conclusions

In conclusion, the current crisis of MDR Gram-negative superbugs coupled with the lack of suitable antibiotics establishes the need for alternative approaches to target them. To address this problem, we resorted to the use of combination of membrane-active molecules (MAMs) and antibiotics. We demonstrated that MAMs re-sensitized tetracycline antibiotics with $>$ 80–1250 fold of reduction in their MIC towards MDR and $bla_{\mathrm{NDM-1}}$ pathogens, *E. coli* and *K. pneumoniae*. Also, the synergistic combination was found to be bactericidal unlike tetracycline antibiotics that are bacteriostatic in activity. Mechanistic studies showed that bacteria had higher uptake of tetracycline in presence of MAMs. Bacteria did not develop resistance to MAMs, unlike to colistin, and moreover MAMs stalled the development of bacterial resistance to antibiotics. MAMs displayed membrane-active properties that had driven the uptake of tetracycline in bacteria. MAMs have strong implications in halting the antibiotic resistance that would otherwise lead to rapid emergence of bacterial resistance. These findings stress the importance of combination approaches of membrane-active molecules and antibiotics to combat MDR Gram-negative superbugs.

Materials and Methods

Ethics Statement

Animal studies were performed according to the protocols approved by Institutional Animal Ethics Committee (IAEC) of Jawaharlal Nehru Centre for Advanced Scientific Research (JNCASR) and National Institute of Veterinary Epidemiology and Disease Informatics (NIVEDI). Acute and sub-chronic toxicity studies were performed at Jawaharlal Nehru Centre for Advanced Scientific Research (JNCASR), Bengaluru (CPCSEA/201) in accordance with institutional ethical guidelines. Infection studies were performed at National Institute of

Veterinary Epidemiology and Disease Informatics (NIVEDI). The animal experiments were approved by the Institutional Animal Ethics Committee (IAEC) of National Institute of Veterinary Epidemiology and Disease Informatics (NIVEDI), Bengaluru (881/GO/ac/05/CPCSEA) and carried out as per the guidelines of Committee for the purpose of Supervision and Experiments on Animals (CPCSEA), Ministry of Environment and Forests, New Delhi, India.

Bacterial Strains, Media, MAMs and Antibiotics

Clinical samples were from Department of Neuromicrobiology, National Institute of Mental Health and Neuro Sciences, Hosur Road, Bangalore 560029, India. Bacterial identification was performed by the Vitek 2 Compact 60 system, bioMerieux, France and Gram-negative bacteria were screened for carbapenem resistance using Kirby- Bauer disc diffusion method (data not shown). *E. coli* (ATCC 25922) was purchased from MTCC (Chandigarh, India). Synthesis of MAMs was as reported previously [23]. Culture media and all the antibiotics were from HIMEDIA and Sigma-Aldrich (India) respectively.

PCR and Gel-electrophoresis

The $bla_{\mathrm{NDM\text{-}1}}$ gene was identified using the primers NDM-F (5′-GGG CAG TCG CTT CCA ACG GT-3′) and NDM-R (5′-GTA GTG CTC AGT GTC GGC AT-3′) (Eurofins Genomics India Pvt. Ltd., Bangalore) which amplified an internal fragment of 475 bp using conventional polymerase chain reaction (PCR). The conditions included an initial denaturation step of 5 min at 94°C, followed by 30 cycles of 30s at 95°C, 30s at 60°C and 30s at 72°C, and then a final extension step of 5 min at 72°C. The PCR products were analyzed in 2% agarose gel, containing 0.05 mg/liter ethidium bromide, at 100V for 1h in 1X Tris Acetate EDTA buffer. A 100 bp DNA ladder was used as a molecular weight marker (SRL Biolit, Mumbai India). Bands were visualized under UV light and an amplified product corresponding to 475 bp was considered as positive.

Antibacterial Assays

Antibacterial activity of the antibiotics, MAMs and colistin was measured and MIC was calculated as per our previously published protocols [23,24]. The combination antibacterial efficacy of MAMs/colistin and antibiotics was measured in nutrient broth using chequerboard assays in the following manner. A solution of 25 μL each of antibiotic and MAMs/colistin was added into each well of a 96 well plate followed by 150 μL of bacterial suspension (~5.0×10^5 CFU mL^{-1}) and incubated at 37°C for 24 h. Bacterial suspension alone and nutrient broth alone served as controls. Each MIC was a result of two independent experiments.

Uptake of tetracycline [6]

The uptake of tetracycline was measured through enhancement in its fluorescence as it enters the bacterial cell membranes. $Bla_{\mathrm{NDM\text{-}1}}$ *E. coli* (R3336) was grown in LB media (OD_{600} ~0.5). The bacteria were harvested, washed and resuspended in 10 mM HEPES (4-(2-hydroxyethyl)-1-piperazineethanesulfonic acid) buffer (pH = 7.2). CCCP and nigericin were dissolved in dimethyl sulfoxide (DMSO) and diluted further in 10 mM HEPES. Always, a freshly made tetracycline solution in water was used owing to its low solubility. All the other test compounds were dissolved in water at 4 mg mL^{-1} and diluted further in HEPES. A bacterial suspension of 100 μl in HEPES containing the test drugs at required concentration (100 μl) was taken for the fluorescence measurements in a Corning 96 well black plate with clear bottom. The fluorescence of tetracycline was monitored using a Tecan InfinitePro series M200 Microplate Reader

at the excitation of 405 nm and emission was collected at 535 nm at room temperature for 30 min. The readout is reported as Tet Fluorescence (a.u.) which is the relative fluorescence calculated by subtracting the background from the fluorescence of bacteria containing samples. The fluorescence of tetracycline alone or in presence of MAMs without the bacteria was used as the background. We found no quenching of the fluorescence of tetracycline in presence of MAMs compared to tetracycline alone without bacteria.

Cytoplasmic Membrane Depolarization Assay

Mid-log phase *bla*$_{NDM-1}$ *E. coli* (R3336) or *E. coli* (ATCC 25922) cells were harvested, washed with 5 mM HEPES and 5 mM glucose and resuspended in 5 mM glucose, 5 mM HEPES buffer and 100 mM KCl solution in 1:1:1 ratio (~10^{8-9} CFU mL^{-1}). Measurements were made in a Corning 96 well black plate with clear bottom with 150 μl of bacterial suspension and 2 μM of DiSC$_3$(5). 0.2 mM of EDTA was used to permeabilize the outer membrane and allow the dye uptake. The fluorescence of the dye was monitored using a Tecan InfinitePro series M200 Microplate Reader at excitation wavelength of 622 nm and emission wavelength of 670 nm. Dye uptake, and resultant self quenching, was modulated by the membrane potential. After reaching the maximum uptake of the dye by bacteria, which was indicated by a minimum in dye fluorescence (after 60 min), the polymer solution was added to the cells, and the decrease in potential was monitored by increase in fluorescence for further 30 min. All the other test compounds dissolved in water at 4 mg mL^{-1} and DiSC$_3$(5) dissolved in DMSO were further diluted in the above 5 mM glucose, 5 mM HEPES buffer and 100 mM KCl solution in 1:1:1 ratio. A control without the polymers served as negative control.

Cytoplasmic Membrane Permeabilization Assay

Mid-log phase *E. coli* (ATCC 25922) or *bla*$_{NDM-1}$ *E. coli* (R3336) cells were harvested, washed, and resuspended in HEPES buffer of pH 7.2 (~10^{8-9} CFU mL^{-1}). Then, 150 μl of bacterial suspension, 10 μM propidium iodide (PI) and polymer solution were added to the cells in a Corning 96 well black plate with clear bottom. Stock solutions of PI and the polymers were made in water and further diluted in HEPES. Excitation wavelength of 535 nm and emission wavelength of 617 nm were used. The uptake of PI was measured using a Tecan InfinitePro series M200 Microplate Reader by the increase in fluorescence of PI for 30 min as a measure of membrane permeabilization.

Bactericidal time-kill kinetics

Briefly, *bla*$_{NDM-1}$ *E. coli* R3336 was grown in nutrient broth at 37°C for 6 h. Test compounds were inoculated with the aliquots of bacteria resuspended in fresh media at ~1.8×10^5 CFU mL^{-1}. After specified time intervals (0, 1, 2, 3, 6, and 24 h), 20 μL aliquots were serially diluted 10 fold in 0.9% saline, plated on sterile MacConkey agar plates and incubated at 37°C overnight. The viable colonies were counted the next day and represented as $\log_{10}$ (CFU mL^{-1}).

Drug Resistance study [24]

In brief, the first MIC determination of MAMs, tetracycline antibiotics and MAMs + tetracycline antibiotics against both *bla*$_{NDM-1}$ *E. coli* R3336 and *E. coli* (ATCC 25922) was performed as described above (Antibacterial Assays). Bacteria from triplicate wells at the concentration of one-half MIC were removed and used to prepare the bacterial dilution (~5.0×10^5 CFU mL^{-1}) for the next experiment. These bacterial suspensions were then used to perform the

antibacterial assay that was repeated for 32 passages. The fold of increase in MIC was determined by normalizing the MIC at passage n to that of the first passage (MIC_n/MIC_1).

Animal Studies

The mice were housed in individually ventilated cages (IVC) maintained with controlled environment as per the standards. They are housing—pathogen free conventional caging system, bedding material—Corn Cob. The husbandry conditions:-Light: dark cycle- 12:12 hours, Animal Room Temp: 22 ± 2°C, Relative humidity: 30–40%, Access to feed and water: *ad libitum* and Water: RO Water. Animals were randomly selected, marked to permit individual identification and kept in their cages for at least 5 days before the experiment to allow for acclimatization to the experimental conditions. Animal handling and experimentation protocols were followed according to OECD Guidelines for the Testing of Chemicals (OECD 425). All care was taken to cause no pain to the animals. Humane endpoints were used to avoid unnecessary distress and suffering in animals following an experimental intervention that would lead to death. All sections of this report adhere to the ARRIVE Guidelines for reporting animal research. A completed ARRIVE guidelines checklist is included in S1 Checklist.

In-vivo systemic toxicity studies

The experimentation protocols for the determination of dosage, number of animals per groups etc. were followed according to the OECD Guidelines for the Testing of Chemicals (OECD 425). Female Balb/c mice (6–8 weeks, 18–22 g) were used for systemic toxicity studies. Mice were randomized into control and test groups with 5 mice per group. Control groups received 200 μL of sterilized PBS (pH = 7.4). Different doses (5.5, 17.5, 55 and 175 mg kg^{-1}) of the test drugs were used as per the OECD guidelines. 200 μL of the test drug solution in sterilized PBS (pH = 7.4) was injected into each mouse (5 mice per group) through intravenous (i.v.) (tail vein) route of administration. The mice in the high dose group (175 mg kg^{-1}) immediately post-injection of the drug, showed clinical signs of tremors, recumbency, sever distress and convulsions, which were indicative of the impending death or moribund condition. Therefore, some which were in moribund condition were humanely euthanized using Isoflurane (Halothane) inhalant anaesthetic. For the intraperitoneal (i.p.) and subcutaneous (s.c.) (over the flank) routes of administration, the high dose (175 mg kg^{-1}) was not injected to reduce the animal lethality. All the mice were monitored for 14 days post-treatment. Different routes of administration were used to find out the best method of administration of test drugs with minimal pain or lethality to the animals. The animals were closely monitored for the first 30 min to 4 hr for the first 24 hours of the initiation of the experiment. And then onwards, they were monitored daily for 14 days. During the observation period of 14 days, no onset of abnormality was found. The 50% lethal dose (LD_{50}) was estimated according to the up- and-down method [32]. The remaining animals, post-experimentation, were euthanized using the same procedure.

In-vivo sub-chronic toxicity studies

Parameters analyzed in subchronic toxicity studies were chosen as per the FDA guidelines of Subchronic Toxicity Study in Rodents. Female Balb/c mice (6–8 weeks, 18–22 g) were used for both acute and subchronic toxicity studies. 200 μL of the polymer solution in sterilized PBS (pH = 7.4) was injected into each mouse. For evaluating the subchronic toxicity- clinical biochemistry parameters, four groups of 10 mice each were given intravenous (i.v.) injection of MAM1 (20 mice) and MAM2 (20 mice) at a dosage of 2 mg kg^{-1} in 200 μL of sterilized PBS (pH = 7.4) and 10 mice were used for the control group. After 48 h, blood was collected from 30

mice (10 mice for MAM1, 10 mice for MAM2 and 10 mice for control) and analyzed for different parameters like alkaline phosphatase (ALP), creatinine, blood urea nitrogen, and electrolytes like sodium, potassium ions and chloride. Also, after 14 days, blood was collected from the remaining mice and analyzed for the above mentioned parameters. Similarly, all the above parameters were analyzed for the control group as well. The data are expressed as mean ± standard deviation, based on values obtained from 10 mice (n = 10 for the data from this report). Statistical analysis was performed using student's *t-test*. Differences are considered statistically significant with probability $p < 0.05$. Post experimentation, mice were euthanized using Isoflurane (Halothane) inhalant anaesthetic.

Mouse Neutropenic Thigh Infection Studies

The experiments were performed in female Swiss-Albino mice (4 mice per group) using a mouse neutropenic thigh infection model against $bla_{\text{NDM-1}}$ *E. coli* (R3336) through intraperitoneal (i. p.) injection of test drugs as described previously in detail [24]. Briefly, the mice were rendered neutropenic (about 100 neutrophils mL^{-1}) by injecting two doses of cyclophosphamide intraperitoneally 4 days (150 mg kg^{-1}) and 1 day (100 mg kg^{-1}) before the infection experiment. About 10^5 CFU mL^{-1} of the bacterial inoculum was injected into the thigh. About 1 h after infection, animals were treated intraperitoneally twice (1 h and 12 h post infection) with saline and compounds. At 24 h post first treatment, cohorts of animals were euthanized (using Isoflurane (Halothane)) and the thighs were collected aseptically. The thigh was weighed and placed into about 10 mL of sterile saline and homogenized. The dilutions of the homogenate were plated onto agar plates, which were incubated overnight at about 37°C. The bacterial titer was expressed as $\log_{10}$ CFU/g of thigh weight and expressed as mean ± S.E.M (standard error of mean). P value was calculated using the unpaired Student's *t* test (2 tailed 2 samples assuming equal variances) and a value $P < 0.05$ was considered significant.

Supporting Information

S1 Checklist. Completed "The ARRIVE Guidelines Checklist" for reporting animal data in this manuscript. (PDF)
(PDF)

S1 Text. Supporting files. Figure A. Antibacterial activity of minocycline and MAMs. The combination of MAM1 and minocycline (25 μg mL^{-1} + 3.1 μg mL^{-1}) showed synergistic bactericidal activity whereas MAM1 (25 μg mL^{-1}) alone and minocycline alone (3.1 μg mL^{-1}) were devoid of antibacterial activity against NDM-1 producing *E. coli* R3336. 20 μL of the bacterial suspension was taken and plated on MacConkey agar plates. Images were taken after 24 h incubation. **Figure B. Development of drug-resistance in tetracycline sensitive *E. coli* (ATCC 25922)**. Fold of increase in MICs of MAMs, tetracycline alone and in combination with MAMs after exposure of *E. coli* (ATCC 25922) to sub-MIC concentrations over 32 passages. *E. coli* did not develop resistance to MAMs. More importantly, resistance against tetracycline alone developed very rapidly but did not develop in presence of MAM1. The MICs of individual agents were as follows: tetracycline (1.56 μg mL^{-1}); MAM1 (15.6 μg mL^{-1}), MAM1 + tetracycline (8 + 1) μg mL^{-1}; MAM2 (250 μg mL^{-1}); MAM2 + tetracycline (62.5 + 1) μg mL^{-1}. **Figure C. Mechanistic studies using tetracycline sensitive *E. coli* (ATCC 25922)**. (A) *E. coli* had much faster and higher uptake of tetracycline in presence MAM2 (20, 30, 60 and 100 μg mL^{-1}) than tetracycline alone (100 μg mL^{-1}). (B) MAMs dissipated the membrane potential in *E. coli* as seen in the increase in fluorescence of $DiSC_3(5)$ after addition of MAMs at 25 μg mL^{-1}; (C) Colistin and MAM2 (both at 60 μg mL^{-1}) increased whereas carbonyl cyanide *m*-chlorophenyl

hydrazone, CCCP (5 μg mL^{-1}) decreased the uptake of tetracycline (100 μg mL^{-1}). (D) MAMs caused cytoplasmic membrane permeabilization as seen in the increase in fluorescence of propidium iodide (PI) after addition of MAMs at 25 μg mL^{-1}; Relative fluorescence was calculated by subtracting the fluorescence without the bacteria from the fluorescence of bacteria containing samples. **Figure D. Uptake of tetracycline against bla_{NDM-1} *E. coli* R3336**. (A) Uptake of tetracycline (100 μg mL^{-1}) in presence of the MAM1 and MAM2 (both at 20 μg mL^{-1}) is much higher and faster compared to tetracycline alone as well as in the presence of nigericin and CCCP (both at 10 μg mL^{-1}). **Table A.** Antibacterial activity of conventional antibiotics in presence or absence of MAMs and colistin against MDR and bla_{NDM-1} clinical isolates. **Table B.** Antibacterial activity of MAMs and colistin against MDR and bla_{NDM-1} clinical isolates. **Table C.** Synergistic profiles of tetracycline antibiotics in combination with MAMs or colistin against MDR and bla_{NDM-1} clinical isolates. **Table D.** Effect of MAMs on the liver and kidney functional parameters and balance of electrolytes in the blood of mice 14 days post-treatment. (DOC)

Acknowledgments

We thank Bharat Ratna Prof. C.N.R. Rao FRS for his constant support and encouragement. Haldar acknowledges the Department of Science and Technology (DST), Govt. of India for Ramanujan fellowship [SR/S2/RJN-43/2009]. Uppu thanks UGC for Junior Research Fellowship (JRF). We thank Dr. R. G. Prakash, In-house animal facility at JNCASR for his co-operation in animal experiments.

Author Contributions

Conceived and designed the experiments: DSSMU JH. Performed the experiments: DSSMU GBM VY JEK KP. Analyzed the data: DSSMU JH RR BRS. Contributed reagents/materials/analysis tools: JH RR BRS. Wrote the paper: DSSMU JH.

References

1. Antimicrobial resistance: global report on surveillance. World health Organization. Geneva. 2014. Available: http://www.who.int/drugresistance/documents/surveillancereport/en.pdf. Accessed 2014 May 9.
2. Walsh TR, Weeks J, Livermore DM, Toleman MA (2011) Dissemination of NDM-1 positive bacteria in the New Delhi environment and its implications for human health: an environmental point prevalence study. Lancet Infect Dis 11: 355–362. doi: 10.1016/S1473-3099(11)70059-7 PMID: 21478057
3. Arpin C, Noury P, Boraud D, Coulange L, Manetti A, et al. (2012) NDM-1-Producing *Klebsiella pneumoniae* Resistant to Colistin in a French Community Patient without History of Foreign Travel. Antimicrob Agents Chemother 56: 3432–3434. doi: 10.1128/AAC.00230-12 PMID: 22450982
4. Deng M, Zhu MH, Li JJ, Bi S, Sheng ZK, et al. (2014) Molecular Epidemiology and Mechanisms of Tigecycline Resistance in Clinical Isolates of *Acinetobacter baumannii* from a Chinese University Hospital. Antimicrob Agents Chemother 58: 297–303. doi: 10.1128/AAC.01727-13 PMID: 24165187
5. King AM, Reid-Yu SA, Wang W, King DT, De Pascale G, et al. (2014) Aspergillomarasmine A overcomes metallo-beta-lactamase antibiotic resistance. Nature 510: 503–506. doi: 10.1038/nature13445 PMID: 24965651
6. Ejim L, Farha MA, Falconer SB, Wildenhain J, Coombes BK, et al. (2011) Combinations of antibiotics and nonantibiotic drugs enhance antimicrobial efficacy. Nat Chem Biol 7: 348–350. doi: 10.1038/nchembio.559 PMID: 21516114
7. Worthington RJ, Bunders CA, Reed CS, Melander C (2012) Small molecule suppression of carbapenem resistance in NDM-1 producing *Klebsiella pneumoniae*. ACS Med Chem Lett 3: 357–361. PMID: 22844552
8. Taylor PL, Rossi L, De Pascale G, Wright GD (2012) A forward chemical screen identifies antibiotic adjuvants in *Escherichia coli*. ACS Chem Biol 7: 1547–1555. PMID: 22698393

9. Goldberg K, Sarig H, Zaknoon F, Epand RF, Epand RM, et al. (2013) Sensitization of gram-negative bacteria by targeting the membrane potential. FASEB J 27: 3818–3826. doi: 10.1096/fj.13-227942 PMID: 23733749
10. Ng VW, Ke X, Lee AL, Hedrick JL, Yang YY (2013) Synergistic co-delivery of membrane-disrupting polymers with commercial antibiotics against highly opportunistic bacteria. Adv Mater 25: 6730–6736. doi: 10.1002/adma.201302952 PMID: 24018824
11. Sakoulas G, Rose W, Nonejuie P, Olson J, Pogliano J, et al. (2014) Ceftaroline restores daptomycin activity against daptomycin-nonsusceptible vancomycin-resistant *Enterococcus faecium*. Antimicrob Agents Chemother 58: 1494–1500. doi: 10.1128/AAC.02274-13 PMID: 24366742
12. Velkov T, Roberts KD, Nation RL, Wang J, Thompson PE, et al. (2014) Teaching 'old' polymyxins new tricks: new-generation lipopeptides targeting gram-negative 'superbugs'. ACS Chem Biol 9: 1172–1177. doi: 10.1021/cb500080r PMID: 24601489
13. Harris TL, Worthington RJ, Hittle LE, Zurawski DV, Ernst RK, et al. (2014) Small molecule downregulation of PmrAB reverses lipid A modification and breaks colistin resistance. ACS Chem Biol 9: 122–127. doi: 10.1021/cb400490k PMID: 24131198
14. Morones-Ramirez JR, Winkler JA, Spina CS, Collins JJ (2013) Silver Enhances Antibiotic Activity Against Gram-Negative Bacteria. Sci Transl Med 5: 190ra81. doi: 10.1126/scitranslmed.3006276 PMID: 23785037
15. Pieri C, Borselli D, Di Giorgio C, De Meo M, Bolla JM, et al. (2014) New Ianthelliformisamine Derivatives as Antibiotic Enhancers against Resistant Gram-Negative Bacteria. J Med Chem 57: 4263–4272. doi: 10.1021/jm500194e PMID: 24801877
16. Zhang J, Chen YP, Miller KP, Ganewatta MS, Bam M, et al. (2014) Antimicrobial Metallopolymers and Their Bioconjugates with Conventional Antibiotics against Multidrug-Resistant Bacteria. J Am Chem Soc 136: 4873–4876. doi: 10.1021/ja5011338 PMID: 24628053
17. Pena-Miller R, Laehnemann D, Jansen G, Fuentes-Hernandez A, Rosenstiel P, et al. (2013) When the Most Potent Combination of Antibiotics Selects for the Greatest Bacterial Load: The Smile-Frown Transition. Plos Biol 11: e1001540. doi: 10.1371/journal.pbio.1001540 PMID: 23630452
18. Liu RH, Chen XY, Chakraborty S, Lemke JJ, Hayouka Z, et al. (2014) Tuning the Biological Activity Profile of Antibacterial Polymers via Subunit Substitution Pattern. J Am Chem Soc 136: 4410–4418. doi: 10.1021/ja500367u PMID: 24601599
19. Sgolastra F, Deronde BM, Sarapas JM, Som A, Tew GN (2013) Designing Mimics of Membrane Active Proteins. Acc Chem Res 46: 2977–2987. doi: 10.1021/ar400066v PMID: 24007507
20. Takahashi H, Palermo EF, Yasuhara K, Caputo GA, Kuroda K (2013) Molecular Design, Structures, and Activity of Antimicrobial Peptide-Mimetic Polymers. Macromol Biosci 13: 1285–1299. doi: 10.1002/mabi.201300126 PMID: 23832766
21. Choi S, Isaacs A, Clements D, Liu D, Kim H, et al. (2009) De novo design and in vivo activity of conformationally restrained antimicrobial arylamide foldamers. Proc Natl Acad Sci U S A 106: 6968–6973. doi: 10.1073/pnas.0811818106 PMID: 19359494
22. Niu YH, Padhee S, Wu HF, Bai G, Qiao Q, et al. (2012) Lipo-gamma-AApeptides as a New Class of Potent and Broad-Spectrum Antimicrobial Agents. J Med Chem 55: 4003–4009. doi: 10.1021/jm300274p PMID: 22475244
23. Uppu DSSM, Akkapeddi P, Manjunath GB, Yarlagadda V, Hoque J, et al. (2013) Polymers with tunable side-chain amphiphilicity as non-hemolytic antibacterial agents. Chem Commun 49: 9389–9391. doi: 10.1039/c3cc43751e PMID: 23868724
24. Yarlagadda V, Akkapeddi P, Manjunath GB, Haldar J (2014) Membrane Active Vancomycin Analogues: A Strategy to Combat Bacterial Resistance. J Med Chem 57: 4558–4568. doi: 10.1021/jm500270w PMID: 24846441
25. Clinical Laboratory Standards Institute. Performance Standards for Antimicrobial Susceptibility Testing, Twenty Second Informational Supplement, M100-S22. Vol. 32 No. 3
26. Manchanda V, Rai S, Gupta S, Rautela RS, Chopra R, et al. (2011) Development of TaqMan real-time polymerase chain reaction for the detection of the newly emerging form of carbapenem resistance gene in clinical isolates of *Escherichia coli*, *Klebsiella pneumoniae*, and *Acinetobacter baumannii*. Indian J Med Microbiol 29: 249–253. doi: 10.4103/0255-0857.83907 PMID: 21860104
27. Odds FC (2003) Synergy, antagonism, and what the chequerboard puts between them. J Antimicrob Chemother 52: 1. PMID: 12805255
28. Walsh C (2000) Molecular mechanisms that confer antibacterial drug resistance. Nature 406: 775–781. PMID: 10963607
29. Dockter ME, Trumble WR, Magnuson JA (1978) Membrane lateral phase separations and chlortetracycline transport by *Bacillus megaterium*. Proc Natl Acad Sci U S A 75: 1319–1323. PMID: 418409

30. Yamaguchi A, Ohmori H, Kaneko-Ohdera M, Nomura T, Sawai T (1991) Delta pH-dependent accumulation of tetracycline in *Escherichia coli*. Antimicrob Agents Chemother 35: 53–56. PMID: 2014981
31. Kashket ER (1985) The proton motive force in bacteria: a critical assessment of methods. Annu Rev Microbiol 39: 219–242. PMID: 2998266
32. Lipnick RL, Cotruvo JA, Hill RN, Bruce RD, Stitzel KA, et al. (1995) Comparison of the up-and-down, conventional LD50, and fixed-dose acute toxicity procedures. Food Chem Toxicol 33: 223–231. PMID: 7896233
